# No causal relationship between glucose and inflammatory bowel disease: a bidirectional two-sample mendelian randomization study

**DOI:** 10.1186/s12920-024-01923-6

**Published:** 2024-06-12

**Authors:** JiePeng Cen, Kequan Chen, Ziyan Ni, QiJie Dai, Weipeng Lu, Heqing Tao, Liang Peng

**Affiliations:** Department of Gastroenterology, The First Affiliated Hospital of Guangzhou Medical University, Guangzhou Medical University, Guangzhou, 510120 Guangdong P.R. China

**Keywords:** Inflammatory bowel disease, Ulcerative colitis, Crohn’s disease, Glucose, Mendelian randomization

## Abstract

**Background:**

Association between glucose and inflammatory bowel disease (IBD) was found in previous observational studies and in cohort studies. However, it is not clear whether these associations reflect causality. Thus, this study investigated whether there is such a causal relation between elevated glucose and IBD, Crohn’s disease (CD) and ulcerative colitis (UC).

**Methods:**

We performed a two-sample Mendelian Randomization (MR) with the independent genetic instruments identified from the largest available genome-wide association study (GWAS) for IBD (5,673 cases; 213,119 controls) and its main subtypes, CD and UC. Summarized data for glucose which included 200,622 cases and glycemic traits including HbA1c and type 2 diabetes(T2DM) were obtained from different GWAS studies. Primary and secondary analyses were conducted by preferentially using the radial inverse-variance weighted (IVW) approach. A number of other meta-analysis approach and sensitivity analyses were carried out to assess the robustness of the results.

**Results:**

We did not find a causal effect of genetically predicted glucose on IBD as a whole (OR 0.858; 95% CI 0.649–1.135; *P* = 0.286). In subtype analyses glucose was also suggestively not associated with Crohn’s disease (OR 0.22; 95% CI 0.04-1.00; *P* = 0.05) and ulcerative colitis (OR 0.940; 95% CI 0.628–1.407; *P* = 0.762). In the other direction, IBD and its subtypes were not related to glucose and glycemic traits.

**Conclusions:**

This MR study is not providing any evidence for a causal relationship between genetically predicted elevated glucose and IBD as well as it’s subtypes UC and CD. Regarding the other direction, no causal associations could be found. Future studies with robust genetic instruments are needed to confirm this conclusion.

**Supplementary Information:**

The online version contains supplementary material available at 10.1186/s12920-024-01923-6.

## Introduction

Inflammatory bowel disease, and its major subtypes: Crohn’s disease and ulcerative colitis, involves chronic relapsing episodes of immune-mediated inflammation of the gastrointestinal tract. In addition to intestinal symptoms, such as abdominal pain and diarrhea, IBD patients frequently experience extra-intestinal symptoms like sacroiliitis and dermatitis contusiformis. No less than 200 genetic susceptibility loci have been confirmed so far, revealing the complexity of the mechanisms of IBD pathogenesis [1, 2]. As we have learned in previous studies [2–4], western diet and lifestyle has an association with the prevalence of IBD, since genetic mutations in the IBD susceptibility genes are independent of race, ethnicity, or demography. Western diet is characterized by higher levels of refined sugar, fat, and animal proteins, and it has been linked to increase the incidence of common chronic diseases including hypertension, type 2 diabetes, coronary disease, and cancer. Although we have learned from recent clinical and experimental studies that high-fat diet may cause IBD [4–6], the role of dietary sugars such as glucose in IBD remains unclear. In previous observational studies and in experimental studies, the effect of glucose on IBD has been widely investigated. Some studies postulated an association between glucose and IBD [7–16]. For example, an experimental study have shown that dietary sugars including glucose is positively associated with IBD risk in mice [16]. However, comparative studies on the effect of total carbohydrate intake and total sugar intake on UC or CD risk found no identified associations. By reason of these controversial findings, we decide to perform a MR study to investigate whether there is a causal relationship between glucose and IBD. We also included subtypes of glycemic traits, including HbA1c and T2DM, as previous studies also used these to monitor the changes and elevated levels in blood glucose [17]. 

By using MR analysis, which takes genetic variation as an instrumental variable (IV), causal relationships between exposure and outcome could be concluded. The principle of MR is that these genetic variants are randomly distributed at meiosis, so that we can strengthen the causal inference [18]. In the present study, we performed a two-sample summary data MR analysis to assess the bidirectional associations of glucose and glycemic traits with IBD and its both conditions CD and UC.

## Materials and methods

### Mendelian randomization design

Genetic variations were selected as instrumental variables and applied to two-sample MR analysis. According to the basic principles of MR study, our study should be carried out under three principal assumptions as follows: (1) the instrumental variables should be closely associated with the exposure; (2) the instrumental variables should be independent of any confounding variables that might take part in the way from exposure to outcome; (3) the instrumental variables only affected results through exposure [19]. 

### Genome-wide association analysis

The genome-wide association studies (GWAS) summary statistics of glycemic traits and IBD were searched and extracted from IEU open gwas project (IEU), European Bioinformatics Institute (EBI) and FinnGen. Since all the aforementioned data was already in the public domain, additional ethical approval was no need. In order to reduce the bias caused by ethnically related confounders as far as possible, the genetic background of our study population was limited to European ancestry.

The EBI was used to identify genetic risk variants for glucose. The database included 200,622 individuals with fasting glucose samples. A dataset identifying 17,724 cases of HbA1c cases and 61,714 of T2DM in addition to 593,952 controls was used for subtype analysis. The diagnosis of T2DM was according to the ICD-10 (International Classification of diseases) criteria.

Different from the above, the Finn database included 5,673 cases with an IBD diagnosis and 213,119 controls. Datasets identifying 2,251 cases of UC with 210,300 controls and 5,956 cases of CD with 14,927 controls were used to assess secondary outcomes. The diagnosis of IBD and its subtypes was based on accepted endoscopic, histopathological, and radio­logical criteria.

### SNP selection

In order to avoid linkage disequilibrium, we use a genome-wide significance level of *p* < 5*10^− 8^ and a cutoff algorithm which r^2^ = 0.001 and kb = 10,000 [18]. Prior to our MR analysis, we quantify the strength of the instrument by utilizing the F-statistic: F = R2(*N* − 2)/(1 - R2), where R2 signifies the proportion of the trait’s variance elucidated by the SNP, and N denotes the sample size of the GWAS encompassing SNPs associated with the trait. The calculated results F-statistic > 10 indicate the absence of weak IVs bias and ample strength, ensuring the credibility of the SNPs.

### Statistical analysis

Our analysis involves an inverse variance weighted model (IVW), which is expected to be stable with balanced pleiotropy, and this approach was used to combine Wald estimates of causality for each IV [20]. Several statistical methods were at the same time used to assess causality. Weighted median estimation models, weighted model-based methods, MR-Egger regression models, simple mode, and MR-polytropic residual sum and outliers (MR-PRESSO) were developed to estimate causal associations under different conditions. When half of the IV is valid, the weighted median estimation models examine the median effect of all available SNPs, resulting in unbiased estimates of the effect. When most individual estimates are derived from valid IVs, weighted model-based models obtain robust population causal estimates [21]. The MR-Egger regression model provides a relatively robust estimate, independent of IV validity, and adjusts the results multi-directional by existing levels by regression slope and intercept. Directional multi-tropism was assessed and corrected according to the intercept obtained from the MR-Egger regression model analysis [22]. MR-PRESSO uses global and SNP-specific sum of squares of observed residuals to test for potential outliers, and corrected causal effects are obtained by excluding outliers with *p* < 0.05 in further distortion tests. The Cochrane’s Q statistic was used to assess the heterogeneity of intravenous delivery. A value of *p* < 0.05 was considered to indicate significant heterogeneity, in which case an IVW approach with the multiplicative random-effect model for subsequent analysis. The random-effect model believes that the true effect sizes may differ in different studies, including both the sampling error and the heterogeneity of the true effects sizes. Under this model, the MR IVW method is summed by weighting the effect sizes and taking between-study heterogeneity into account. If there is significant heterogeneity, the random-effect model may be more appropriate [20, 23]. Otherwise, a fixed effects model should be used. IVs were excluded by leave-one-out analysis to judge the stability of the MR Results. However, the methods above-mentioned yielded wide confidence intervals (CI) compared with the IVW approach and were only used as complementary methods. Therefore, the MR-Egger regression model was used in the case of significant pleiotropy, and the MR-PRESSO model was used to detect the final outliers. Otherwise, IVW results were always in a priority position [18]. Based on the causal relationship between 3 glycemic traits and IBD, the more conservative Bonferroni method was utilized to correct for significance results. Before correction, *p* < 0.05 was a significant result, while *p* < 0.016 (0.05/3) was a significant result after Bonferroni correction [24, 25]. In addition, results with *p* < 0.016 were considered as strong evidence for causal relationship, while results with *p* < 0.05 but no lower than 0.016 were considered for potential and weak causal associations [26–29].Causal relationship was presented as odds ratio (OR), 95% confidence interval (CI)and the forest plot. Our bidirectional two-sample MR Analyses were performed by using software R (version 4.3.0,), Rstudio (version 2023.03.0 Build 386) and TwoSampleMR (version 0.5.6) with the MR-PRESSO package (version 1.0.0) and forestplot package (version 3.1.1).

## Results

### Instrumental variable statistical results

Through the above series of screening processes, 13 SNPs related to total IBD, 121 SNPs related to CD, and 6 SNPs related to UC were selected to take part in the MR analyses. There were also 66 SNPs related to total glucose, 12 SNPs related to HbA1c and 118 SNPs related to T2DM for MR analysis, respectively. The F-statistics of the SNPs in our study ranged from 24.5198 to 1624.0697, which are all greater than 10, indicating a strong association between the IVs and the exposure. Details of the SNPs that performed their MR analyses and results of F-statistic were shown in Supplementary materials 5 (Supplementary Tables 1, 2).

### Primary MR analysis of bidirectional associations between IBD and glucose

**Fig. 1:**
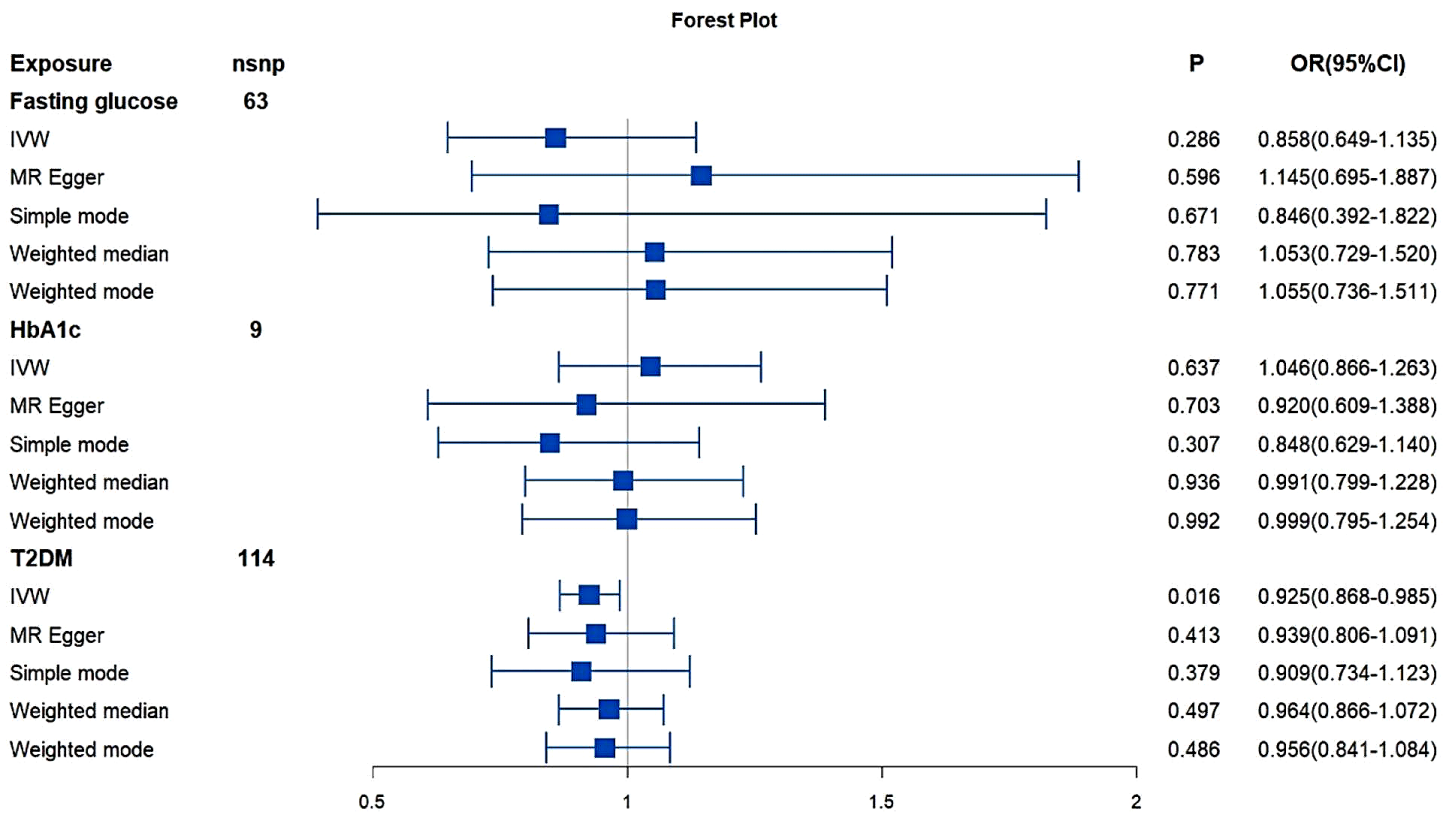
MR results and forest plot where glucose, HbA1c, T2DM as the exposure and IBD as the outcome. MR: Mendelian Randomization; IBD: Inflammatory Bowel Disease; T2DM: type 2 diabetes; IVW: inverse variance weighted; OR: odds ratio; CI: confidence interval

No directional pleiotropy was shown in MR-Egger regression (Intercept = -0.0081; *P* = 0.181) for the influence of glucose on IBD. Significant heterogeneity was not found by Cochran’s Q test (Q = 79.286; *P* = 0.068) and no outliers were identified. The results did not indicate a directly causal effect of glucose on IBD (IVW: OR: 0.858; 95% CI: 0.649–1.135; *P* = 0.286). The table of the MR results and forest plots for each outcome in replication practice were shown in Table 1; Fig. 1.

**Table 1:** MR results where glucose, HbA1c and T2DM as the exposure and IBD as the outcome

Exposure	*P*	OR(95%CI)	*P* for Q-Test	*P* for Intercept
**Fasting glucose**				
IVW	0.286	0.858(0.649–1.135)	0.069	0.182
MR Egger	0.596	1.145(0.695–1.887)		
Simple mode	0.671	0.846(0.392–1.822)		
Weighted median	0.783	1.053(0.729–1.520)		
Weighted mode	0.771	1.055(0.736–1.511)		
**HbA1c**				
IVW	0.637	1.046(0.866–1.263)	0.182	0.508
MR Egger	0.703	0.920(0.609–1.388)		
Simple mode	0.307	0.848(0.629–1.140)		
Weighted median	0.936	0.991(0.799–1.228)		
Weighted mode	0.992	0.999(0.795–1.254)		
**T2DM**				
IVW	0.016	0.925(0.868–0.985)	0.111	0.837
MR Egger	0.413	0.939(0.806–1.091)		
Simple mode	0.379	0.909(0.734–1.123)		
Weighted median	0.497	0.964(0.866–1.072)		
Weighted mode	0.486	0.956(0.841–1.084)		

MR: Mendelian Randomization; IBD: Inflammatory Bowel Disease; T2DM: type 2 diabetes; IVW: inverse variance weighted; OR: odds ratio; CI: confidence interval

**Fig. 2:**
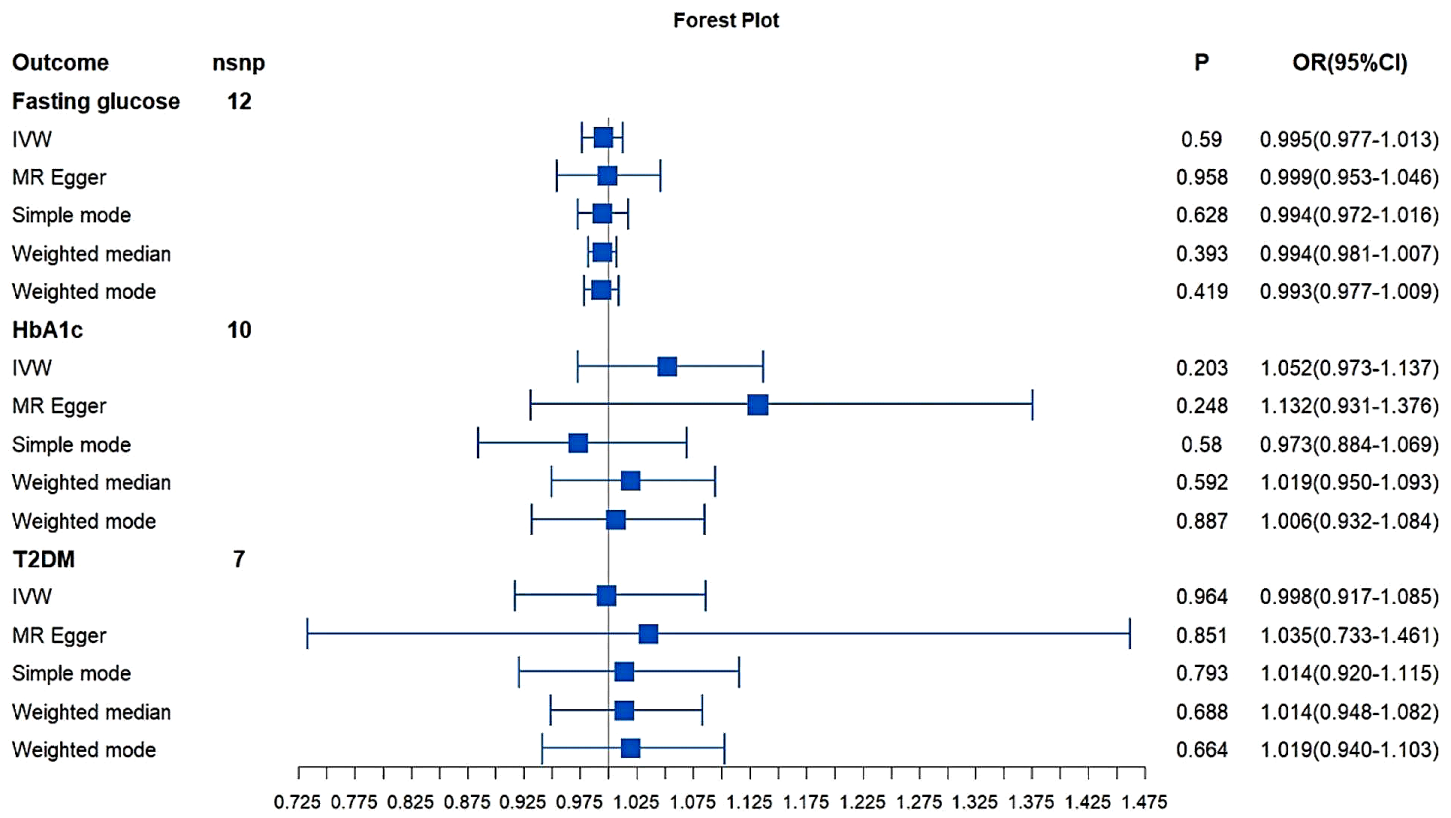
MR results and forest plot where glucose, HbA1c, T2DM as the outcome and IBD as the exposure. MR: Mendelian Randomization; IBD: Inflammatory Bowel Disease; T2DM: type 2 diabetes; IVW: inverse variance weighted; OR: odds ratio; CI: confidence interval

In the other direction, heterogeneity was found by Cochran’s Q test (Q = 46.86; *P* = 2.27*10^− 6^) and an IVW approach with the multiplicative random-effect model was applied to the main analyses. Although, significant directional pleiotropy was not found by MR-Egger regression analysis (Intercept = -0.000628; *P* = 0.867) and genetically predicted IBD was not associated with glucose (OR:0.995;95% CI: 0.977–1.013; *P* = 0.589). The table of the MR results and forest plots for all the outcome in practice were shown in Table 2; Fig. 2. As for the heterogeneity results and leave-one-out plots were presented in Supplementary material 2, 3.

**Table 2:** Mendelian randomization (MR) results where glucose, HbA1c and T2DM as the outcome and IBD as the exposure

Outcome	*P*	OR(95%CI)	*P* for Q-Test	*P* for Intercept
**Fasting glucose**				
IVW	0.590	0.995(0.977–1.013)	2.27*10^− 6^	0.868
MR Egger	0.958	0.999(0.953–1.046)		
Simple mode	0.628	0.994(0.972–1.016)		
Weighted median	0.393	0.994(0.981–1.007)		
Weighted mode	0.419	0.993(0.977–1.009)		
**HbA1c**				
IVW	0.203	1.052(0.973–1.137)	5.96*10^− 3^	0.442
MR Egger	0.248	1.132(0.931–1.376)		
Simple mode	0.580	0.973(0.884–1.069)		
Weighted median	0.592	1.019(0.950–1.093)		
Weighted mode	0.887	1.006(0.932–1.084)		
**T2DM**				
IVW	0.964	0.998(0.917–1.085)	6.20*10^− 3^	0.837
MR Egger	0.851	1.035(0.733–1.461)		
Simple mode	0.793	1.014(0.920–1.115)		
Weighted median	0.688	1.014(0.948–1.082)		
Weighted mode	0.664	1.019(0.940–1.103)		

MR: Mendelian Randomization; IBD: Inflammatory Bowel Disease; T2DM: type 2 diabetes; IVW: inverse variance weighted; OR: odds ratio; CI: confidence interval

### MR analysis of bidirectional associations between IBD and other glycemic traits

The results did not indicate a directly causal effect of HbA1c on IBD (HbA1c: IVW: OR: 1.046; 95% CI: 0.866–1.263; *P* = 0.637). From the MR analysis results of T2DM on IBD (T2DM: IVW: OR: 0.925; 95% CI: 0.868–0.985; *P* = 0.016) we can see that there is a weak and potential causal relationship between genetically predicted T2DM on IBD since the P-value is < 0.05 but no lower than 0.016 [26, 27].

In the order direction, genetically predicted IBD was not associated with glycemic traits (HbA1c: IVW: OR: 1.052; 95% CI: 0.973–1.137; *P* = 0.202; T2DM: IVW: OR: 0.998; 95% CI: 0.917–1.085; *P* = 0.964). The MR estimates from different methods of assessing the causal effect between IBD and other glycemic traits were presented in Tables 1 and 2, and the forest plots were shown in Figs. 1 and 2.

### Secondary MR analysis

#### Effect of glucose on IBD’s main subtypes

Directional pleiotropy was not found in MR-Egger regression for the influence of glucose on CD or UC. Significant heterogeneity was found by Cochran’s Q test but no outliers were identified. Hence, multiplicative random-effect models were applied in the IVW analysis. The results did not indicate a directly causal effect of glucose on UC. (UC: IVW: OR: 0.940; 95% CI: 0.628–1.407; *P* = 0.762; CD: IVW: OR: 0.214; 95% CI: 0.046–0.999; *P* = 0.0498). Although our MR analysis’s results did indicate the causality of glucose on CD, we presume that the causal relationship is potential so that further investigation is needed, since the P-value is < 0.05 but no lower than 0.016 [26, 27]. 

#### Effect of IBD’s main subtypes on glucose

Analyses of UC and CD on glucose did not show significant heterogeneity, but directional pleiotropy was found. As a result, multiplicative random-effect models were applied in the IVW analysis. No significant association emerged between UC, CD and glucose (UC: IVW: OR: 1.324; 95% CI: 0.974–2.006; *P* = 0.185; CD: IVW: OR: 0.998; 95% CI: 0.991–1.004; *P* = 0.479). All the MR results and the forest plots from different methods of assessing the causal effect between IBD’s Main Subtypes and glucose were shown in Supplementary materials 1. In addition, the heterogeneity plots and leave-one-out plots were presented in Supplementary material 2, 3.

As for the causal effect between glycemic traits and IBD’s main subtypes, please check out the supplementary material for more details. (Supplementary materials 1, 2, 3).

## Discussion

It was found in this study that no causal relationship was detected between the glycemic traits and IBD. The sensitive analysis, including the pleiotropic test and the leave-one-out analysis, confirmed the robustness and reliability of the relationship. Supplementary MR methods also proved the validity of the results (Supplementary materials 1, 2, 3).

The effect of glucose, as a major component of the western diet, on IBD has been widely investigated. Some previous epidemiological cohort studies, comparative study [7–15] and experimental studies [16] postulated an association between glucose and IBD. For example, an experimental study [16] suggests that intake of simple sugars predisposes to colitis and enhances its pathogenesis via modulation of gut microbiota in mice. From this study it seems like there is a causal relationship between IBD and glucose, but this study in mice has to be further verified through the conclusion of the case-control study in human.

In a cohort study from Taiwan [7] with a total of 364,689 participants, a reduced risk of IBD is consistently observed in patients with type 2 diabetes mellitus who have been treated with metformin. Metformin may reduce IBD risk via changes in the metabolism of glucose, since metformin can reduce blood glucose level, as a result, the association between glucose and IBD needs to be further investigated. Taking all the evidence in this study together, metformin may reduce IBD risk via multiple mechanisms by targeting insulin resistance, changes in the gut microbiota and metabolism of nutrients, an energy shift to ketone bodies, inhibition of inflammation and modulating immunity. The metformin can exert a complicated and significant effect on the association between glucose and intestine-related disorders. Therefore, there is not enough evidence in this passage to prove the causal relationship between glucose and IBD.

In a multi-center prospective study [13], any associations were not found between total dietary carbohydrate or sugar intake and the odds of developing CD or UC, which come to the same conclusion with our MR analyses. The previous retrospective case–control studies have reported conflicting findings [30, 31]. These may result from methodological errors including selection and recall biases. Compared with prospective study, patients often have difficulties reporting their pre-illness diet in retrospective studies, particularly if the illness was diagnosed many years ago.

Since we have learned from the previous studies [32, 33] that there is association between glucose and gut micro biota, and there is a relationship between gut micro biota and IBD [34, 35], we plan to conduct additional analyses to investigate potential factors like gut micro biota that could mediate the relationship between glucose and IBD. This will include a more rigorous examination of the IVs to ensure their validity and a detailed analysis to calculate the causal effects exerted by these potential mediating effects.

Exploring the causal effect of glucose on IBD was the key point in the investigation. Genetic variants were only instrumental variables to perform the analysis. It is difficult to accurately detect and monitor the intake value of glucose, which is an advantage of a Mendelian randomization analysis. In a traditional epidemiological study, dietary and other habits could have enormous impacts on the relation of glucose with IBD. However, our study explored the IBD risk variation based on glucose level determined by genetic variants. The MR analysis was designed to avoid traditional confounders, such as dietary and other habits, by introducing instrumental variables, and to monitor their interference by sensitivity analysis. So, the influences of dietary and other habits could be balanced and would not affect the results.

The remain strengths of this MR study are listed as follows. Firstly, the design of the research was based on three principal instrumental variable assumptions and conformed to the checklist for performing MR investigations. Thus, the conclusions drawn in this study were reasonable and could be trusted. Secondly, the two large-scale GWASs were both obtained from European ancestries, which allowed the bias of population stratification to be avoided. Thirdly, a total of five MR analysis methods were applied to the evaluation of the consistency of causal effects.

Meanwhile, some weaknesses cannot be ignored in this study. Firstly, both FINN, EBI and IIBDGC participants were Europeans, and, thus, the generalizability of the results was limited. Secondly, the IBD patients were from different medical centers, and the differences in diagnosis methods, information acquisition and data processing might bias the results. Thirdly, this study only provided robust and reliable evidence for the effect of glucose on IBD risk. The effect of glucose on established IBD patients has not yet been explored, and the effect of different elevated blood glucose level on IBD risk has not yet been explored, too.

## Conclusion

This MR study ruled out the causal relationship between IBD and glucose, suggesting that therapy aim at glucose or T2DM might not work for IBD and vice versa.

### Electronic supplementary material

Below is the link to the electronic supplementary material.

Supplementary Material 1

Supplementary Material 2

Supplementary Material 3

Supplementary Material 4

Supplementary Material 5

## Data Availability

The datasets presented in this study can be found in online repositories. The names of the repository/repositories can be found in the article. The GWAS summary statistics for CD, T2DM and glucose is available on the website https://www.ebi.ac.uk/. The GWAS summary statistics for IBD and UC is available in the FinnGen website (https://www.finngen.fi/en). The GWAS summary statistics for HbA1c is available on the website https://gwas.mrcieu.ac.uk/.

## Abbreviations

CD: Crohn’s Disease
CI: Confidence Intervals
EBI: European Bioinformatics Institute
GWAS: Genome-wide Association Study
IBD: Inflammatory Bowel Disease
ICD: International Classification Of Diseases
IEU: IEU Open Gwas Project
IV: Instrumental Variable
IVW: Inverse-Variance Weighted
MR: Mendelian Randomization
MR-PRESSO: MR-polytropic Residual Sum And Outliers
OR: Odds Ratio
T2DM: Type 2 Diabetes
UC: Ulcerative Colitis
