# Supplementary material for: No causal relationship between glucose and inflammatory bowel disease: a bidirectional two-sample mendelian randomization study
Source: BMC Med Genomics. 2024 Jun 12;17:159. doi: 10.1186/s12920-024-01923-6 (PMC11167808; doi:10.1186/s12920-024-01923-6)
Supplement: Supplementary file 1 — Supplementary Material 1 [file 12920_2024_1923_MOESM1_ESM.doc]

***Supplementary Material 1:*** ***MR Results and Forest Plots***

**No Causal Relationship Between Glucose and** **Inflammatory Bowel Disease: A Bidirectional Two-Sample Mendelian Randomization Study**

JiePeng Cen, MD^1^†, Kequan Chen, MD^1^†, Ziyan Ni, MD^1^†, QiJie Dai^1^, MD, Weipeng Lu^1^, MD, Heqing Tao^1^, MD and Liang Peng^1^, MD

**Corresponding author:** Liang Peng: [wsfirefly@126.com](mailto:wsfirefly@126.com)

**1 Supplementary Figures**


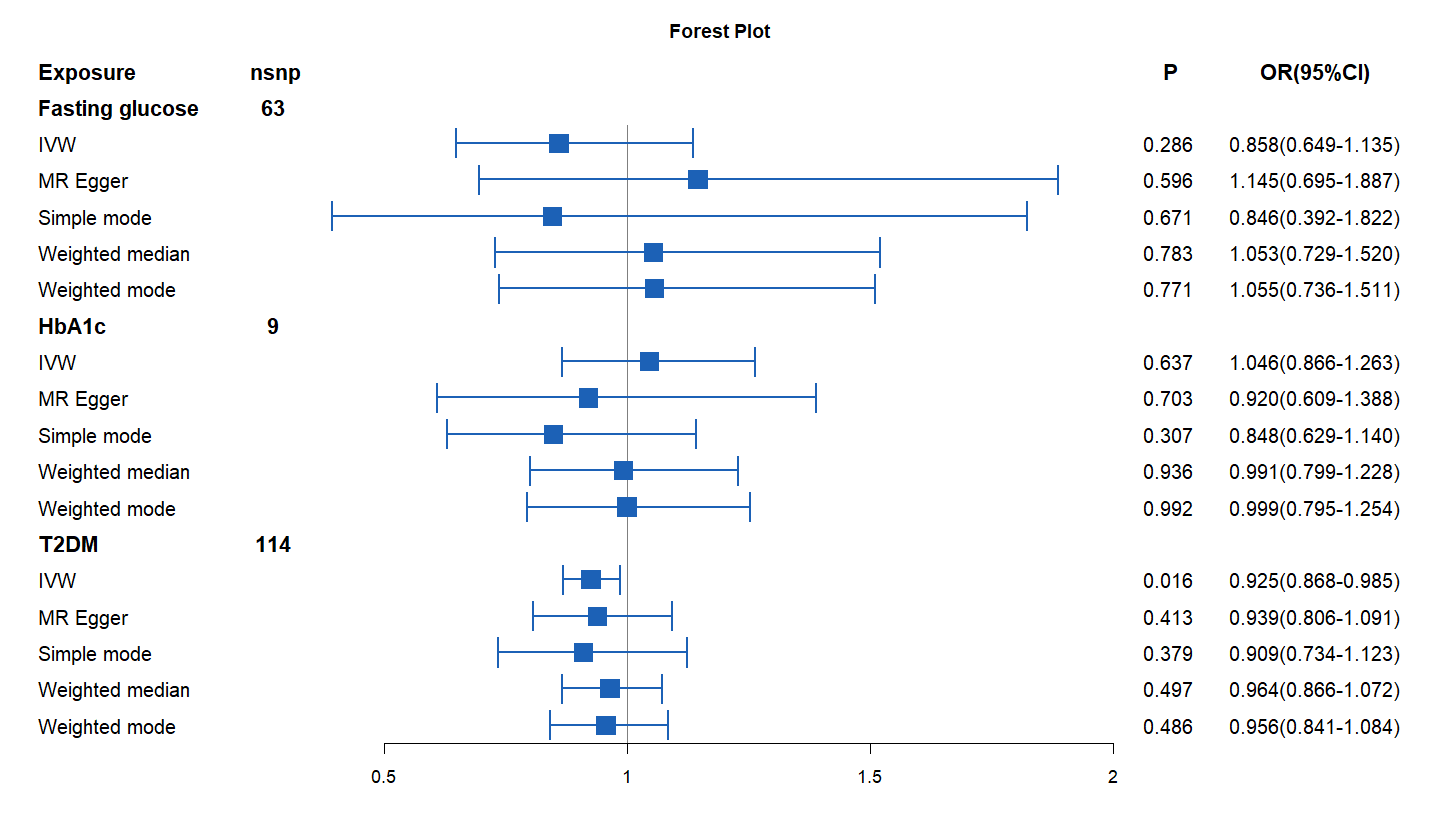


Figure 1.1 MR results and forest plot where glucose, HbA1c, T2DM as the exposure and IBD as the outcome. IVW, inverse-variance weighted; T2DM, type 2 diabetes. OR, odds ratio; The error bar indicates the 95% confidence interval.


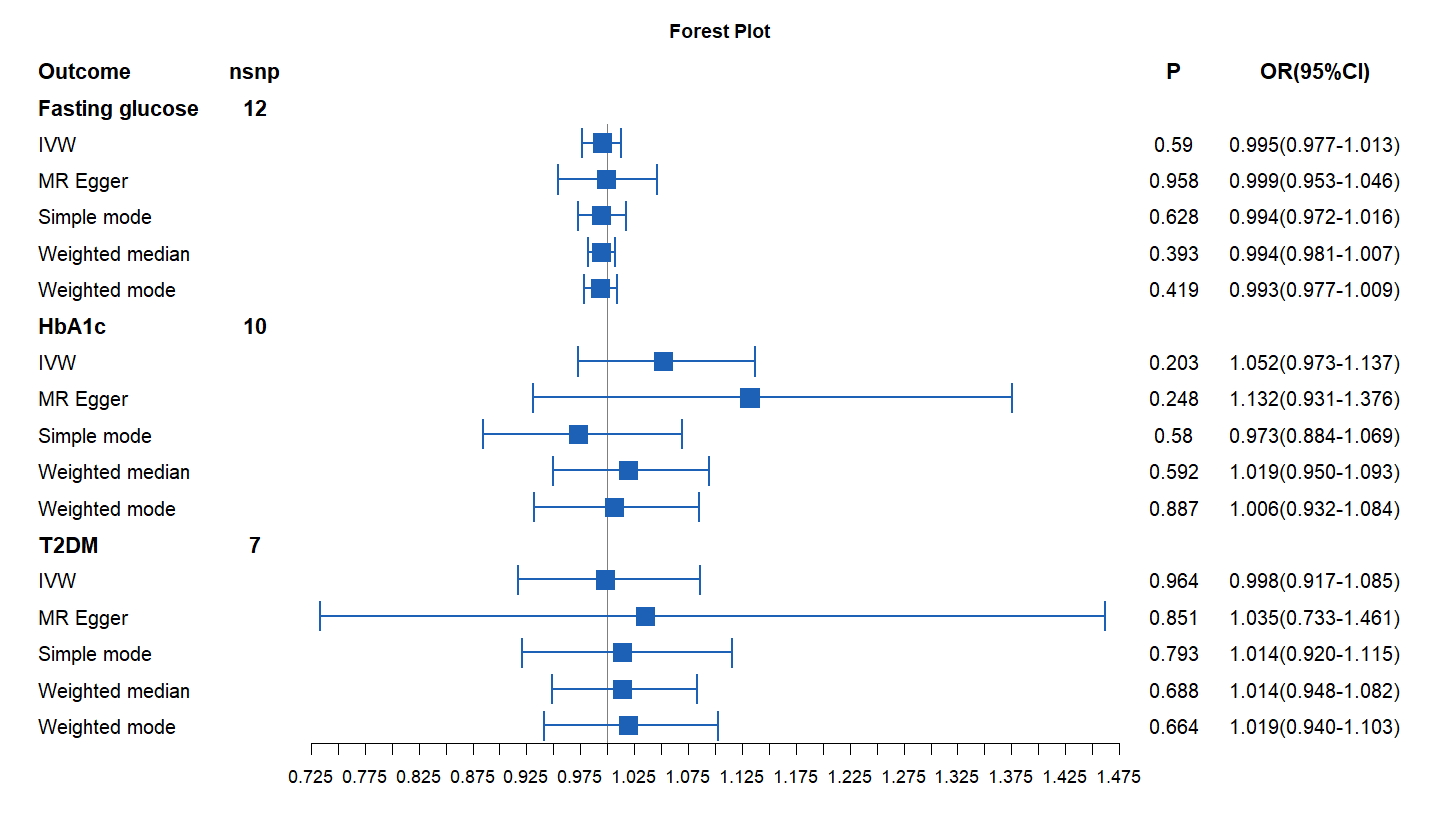


Figure 1.2 MR results and forest plot where glucose, HbA1c, T2DM as the outcome and IBD as the exposure. IVW, inverse-variance weighted; T2DM, type 2 diabetes. OR, odds ratio; The error bar indicates the 95% confidence interval.


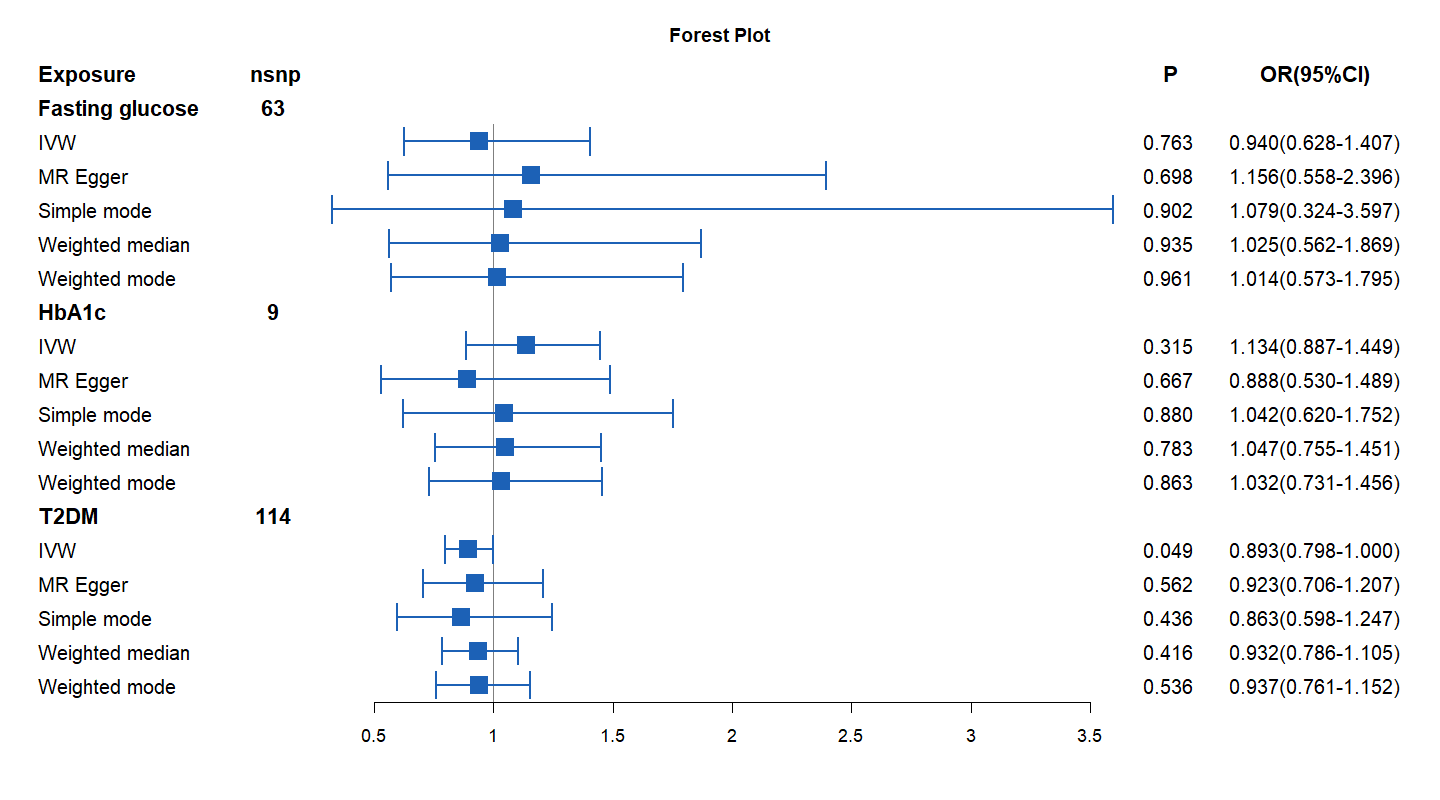


Figure 1.3 MR results and forest plot where glucose, HbA1c, T2DM as the exposure and UC as the outcome. IVW, inverse-variance weighted; T2DM, type 2 diabetes. OR, odds ratio; The error bar indicates the 95% confidence interval.


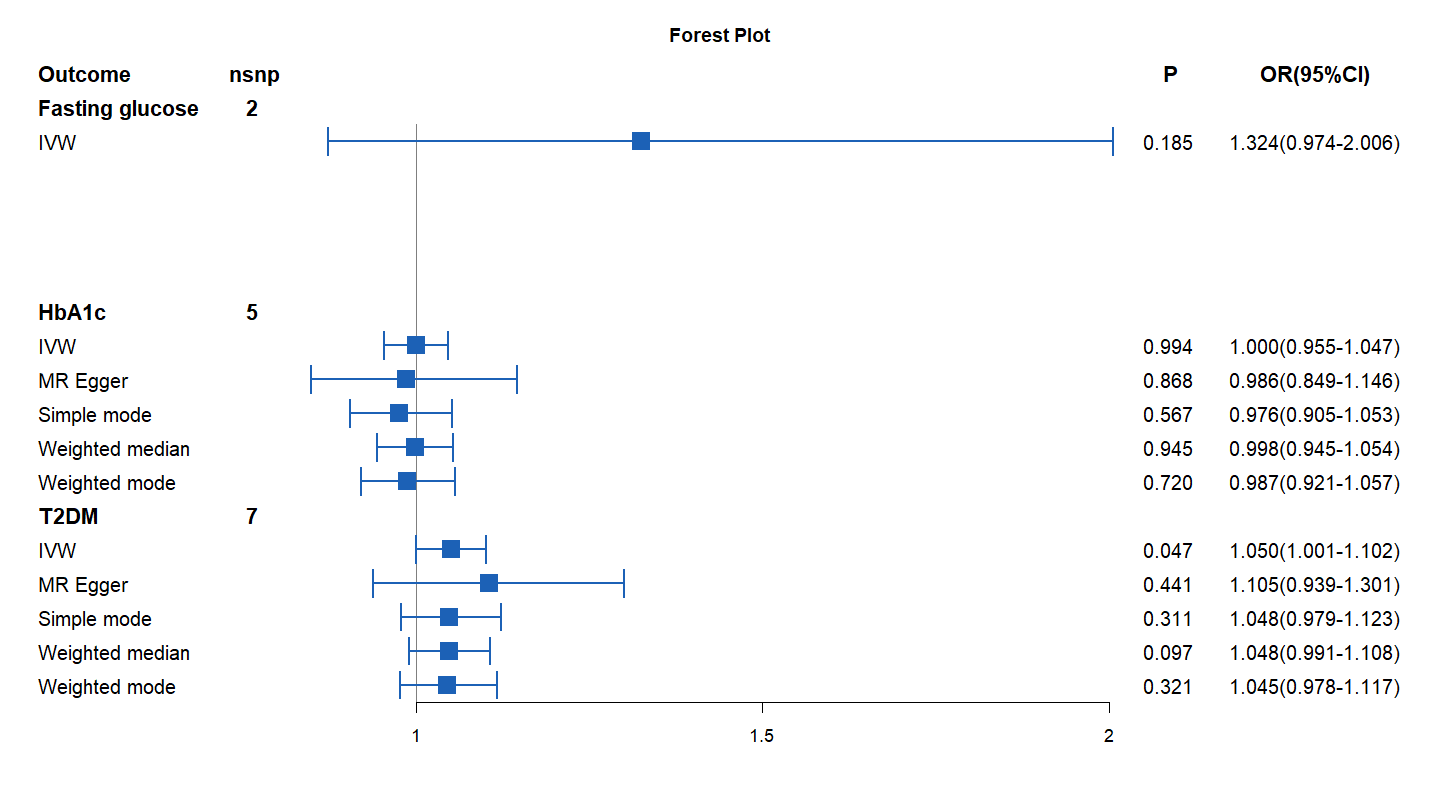


Figure 1.4 MR results and forest plot where glucose, HbA1c, T2DM as the outcome and UC as the exposure. IVW, inverse-variance weighted; T2DM, type 2 diabetes. OR, odds ratio; The error bar indicates the 95% confidence interval.


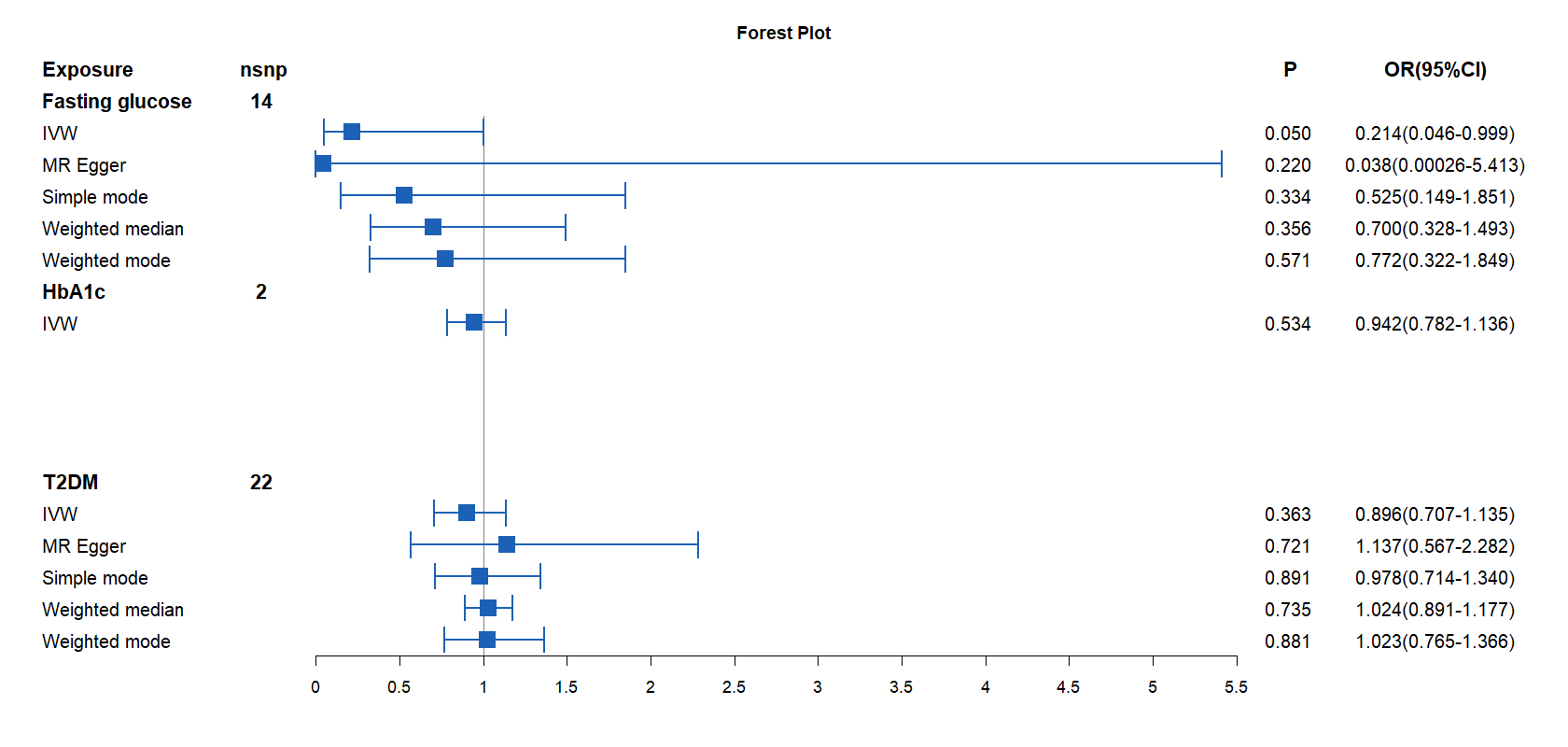


Figure 1.5 MR results and forest plot where glucose, HbA1c, T2DM as the exposure and CD as the outcome. IVW, inverse-variance weighted; T2DM, type 2 diabetes. OR, odds ratio; The error bar indicates the 95% confidence interval.


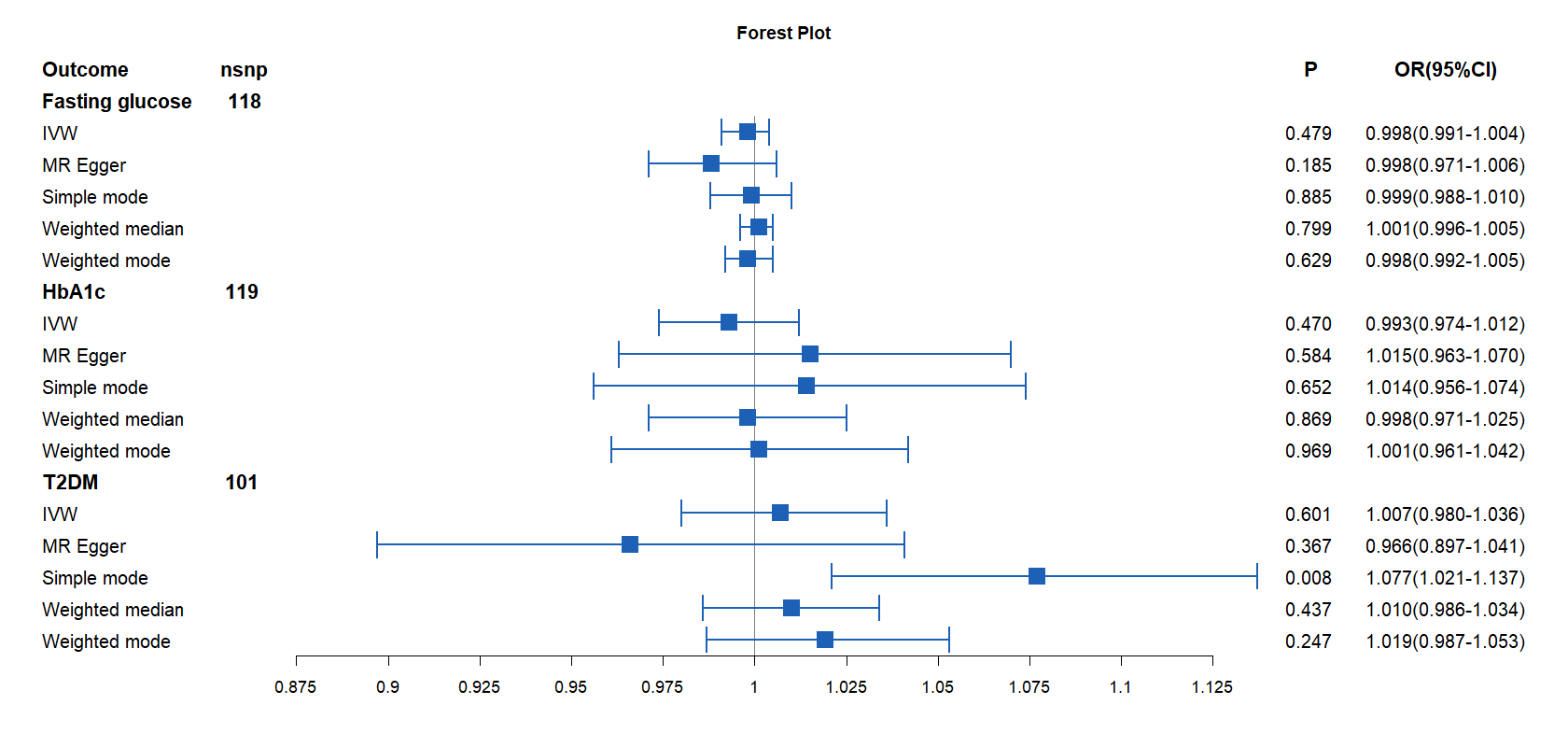


Figure 1.6 MR results and forest plot where glucose, HbA1c, T2DM as the outcome and CD as the exposure. IVW, inverse-variance weighted; T2DM, type 2 diabetes. OR, odds ratio; The error bar indicates the 95% confidence interval.

**2 Supplementary Tables**

Table 2.1 MR results where glucose, HbA1c, T2DM as the exposure and IBD as the outcome.

| **Exposure** | **P** | **OR (95%CI)** | **P for Q-Test** | **P for Intercept** |
| --- | --- | --- | --- | --- |
| **Fasting glucose** |  |  |  |  |
| IVW | 0.286 | 0.858(0.649-1.135) | 0.069 | 0.182 |
| MR Egger | 0.596 | 1.145(0.695-1.887) |  |  |
| Simple mode | 0.671 | 0.846(0.392-1.822) |  |  |
| Weighted median | 0.783 | 1.053(0.729-1.520) |  |  |
| Weighted mode | 0.771 | 1.055(0.736-1.511) |  |  |
| **HbA1c** |  |  |  |  |
| IVW | 0.637 | 1.046(0.866-1.263) | 0.182 | 0.508 |
| MR Egger | 0.703 | 0.920(0.609-1.388) |  |  |
| Simple mode | 0.307 | 0.848(0.629-1.140) |  |  |
| Weighted median | 0.936 | 0.991(0.799-1.228) |  |  |
| Weighted mode | 0.992 | 0.999(0.795-1.254) |  |  |
| **T2DM** |  |  |  |  |
| IVW | 0.016 | 0.925(0.868-0.985) | 0.111 | 0.837 |
| MR Egger | 0.413 | 0.939(0.806-1.091) |  |  |
| Simple mode | 0.379 | 0.909(0.734-1.123) |  |  |
| Weighted median | 0.497 | 0.964(0.866-1.072) |  |  |
| Weighted mode | 0.486 | 0.956(0.841-1.084) |  |  |

T2DM, type 2 diseases; OR, odds ratio; IVW, inverse-variance weighted; Q-test: Cochran’s Q statistic;

Table 2.2 MR results where glucose, HbA1c, T2DM as the outcome and IBD as the exposure.

| **Outcome** | **P** | **OR (95%CI)** | **P for Q-Test** | **P for Intercept** |
| --- | --- | --- | --- | --- |
| **Fasting glucose** |  |  |  |  |
| IVW | 0.590 | 0.995(0.977-1.013) | 2.27*10^-6^ | 0.868 |
| MR Egger | 0.958 | 0.999(0.953-1.046) |  |  |
| Simple mode | 0.628 | 0.994(0.972-1.016) |  |  |
| Weighted median | 0.393 | 0.994(0.981-1.007) |  |  |
| Weighted mode | 0.419 | 0.993(0.977-1.009) |  |  |
| **HbA1c** |  |  |  |  |
| IVW | 0.203 | 1.052(0.973-1.137) | 5.96*10^-3^ | 0.442 |
| MR Egger | 0.248 | 1.132(0.931-1.376) |  |  |
| Simple mode | 0.580 | 0.973(0.884-1.069) |  |  |
| Weighted median | 0.592 | 1.019(0.950-1.093) |  |  |
| Weighted mode | 0.887 | 1.006(0.932-1.084) |  |  |
| **T2DM** |  |  |  |  |
| IVW | 0.964 | 0.998(0.917-1.085) | 6.20*10^-3^ | 0.837 |
| MR Egger | 0.851 | 1.035(0.733-1.461) |  |  |
| Simple mode | 0.793 | 1.014(0.920-1.115) |  |  |
| Weighted median | 0.688 | 1.014(0.948-1.082) |  |  |
| Weighted mode | 0.664 | 1.019(0.940-1.103) |  |  |

T2DM, type 2 diseases; OR, odds ratio; IVW, inverse-variance weighted; Q-test: Cochran’s Q statistic;

Table 2.3 MR results where glucose, HbA1c, T2DM as the exposure and UC as the outcome.

| **Exposure** | **P** | **OR (95%CI)** | **P for Q-Test** | **P for Intercept** |
| --- | --- | --- | --- | --- |
| **Fasting glucose** |  |  |  |  |
| IVW | 0.763 | 0.940(0.628-1.407) | 0.258 | 0.506 |
| MR Egger | 0.698 | 1.156(0.558-2.396) |  |  |
| Simple mode | 0.902 | 1.079(0.324-3.597) |  |  |
| Weighted median | 0.935 | 1.025(0.562-1.869) |  |  |
| Weighted mode | 0.961 | 1.014(0.573-1.795) |  |  |
| **HbA1c** |  |  |  |  |
| IVW | 0.315 | 1.134(0.887-1.449) | 0.442 | 0.328 |
| MR Egger | 0.667 | 0.888(0.530-1.489) |  |  |
| Simple mode | 0.880 | 1.042(0.620-1.752) |  |  |
| Weighted median | 0.783 | 1.047(0.755-1.451) |  |  |
| Weighted mode | 0.863 | 1.032(0.731-1.456) |  |  |
| **T2DM** |  |  |  |  |
| IVW | 0.049 | 0.893(0.798-1.000) | 2.29*10^-4^ | 0.790 |
| MR Egger | 0.562 | 0.923(0.706-1.207) |  |  |
| Simple mode | 0.436 | 0.863(0.598-1.247) |  |  |
| Weighted median | 0.416 | 0.932(0.786-1.105) |  |  |
| Weighted mode | 0.536 | 0.937(0.761-1.152) |  |  |

T2DM, type 2 diseases; OR, odds ratio; IVW, inverse-variance weighted; Q-test: Cochran’s Q statistic;

Table 2.4 MR results where glucose, HbA1c, T2DM as the outcome and UC as the exposure.

| **Outcome** | **P** | **OR (95%CI)** | **P for Q-Test** | **P for Intercept** |
| --- | --- | --- | --- | --- |
| **Fasting glucose** |  |  |  |  |
| IVW | 0.185 | 1.324(0.974-2.006) | 5.13*10^-6^ |  |
| MR Egger |  |  |  |  |
| Simple mode |  |  |  |  |
| Weighted median |  |  |  |  |
| Weighted mode |  |  |  |  |
| **HbA1c** |  |  |  |  |
| IVW | 0.994 | 1.000(0.955-1.047) | 0.794 | 0.860 |
| MR Egger | 0.868 | 0.986(0.849-1.146) |  |  |
| Simple mode | 0.567 | 0.976(0.905-1.053) |  |  |
| Weighted median | 0.945 | 0.998(0.945-1.054) |  |  |
| Weighted mode | 0.720 | 0.987(0.921-1.057) |  |  |
| **T2DM** |  |  |  |  |
| IVW | 0.047 | 1.050(1.001-1.102) | 0.776 | 0.635 |
| MR Egger | 0.441 | 1.105(0.939-1.301) |  |  |
| Simple mode | 0.311 | 1.048(0.979-1.123) |  |  |
| Weighted median | 0.097 | 1.048(0.991-1.108) |  |  |
| Weighted mode | 0.321 | 1.045(0.978-1.117) |  |  |

T2DM, type 2 diseases; OR, odds ratio; IVW, inverse-variance weighted; Q-test: Cochran’s Q statistic;

Table 2.5 MR results where glucose, HbA1c, T2DM as the exposure and CD as the outcome.

| **Exposure** | **P** | **OR (95%CI)** | **P for Q-Test** | **P for Intercept** |
| --- | --- | --- | --- | --- |
| **Fasting glucose** |  |  |  |  |
| IVW | 0.050 | 0.214(0.046-0.999) | 2.58*10^-36^ | 0.483 |
| MR Egger | 0.220 | 0.038(0.00026-5.413) |  |  |
| Simple mode | 0.334 | 0.525(0.149-1.851) |  |  |
| Weighted median | 0.356 | 0.700(0.328-1.493) |  |  |
| Weighted mode | 0.571 | 0.772(0.322-1.849) |  |  |
| **HbA1c** |  |  |  |  |
| IVW | 0.534 | 0.942(0.782-1.136) | 0.653 |  |
| MR Egger |  |  |  |  |
| Simple mode |  |  |  |  |
| Weighted median |  |  |  |  |
| Weighted mode |  |  |  |  |
| **T2DM** |  |  |  |  |
| IVW | 0.363 | 0.896(0.707-1.135) | 1.64*10^-33^ | 0.483 |
| MR Egger | 0.721 | 1.137(0.567-2.282) |  |  |
| Simple mode | 0.891 | 0.978(0.714-1.340) |  |  |
| Weighted median | 0.735 | 1.024(0.891-1.177) |  |  |
| Weighted mode | 0.881 | 1.023(0.765-1.366) |  |  |

T2DM, type 2 diseases; OR, odds ratio; IVW, inverse-variance weighted; Q-test: Cochran’s Q statistic;

Table 2.6 MR results where glucose, HbA1c, T2DM as the outcome and CD as the exposure.

| **Outcome** | **P** | **OR (95%CI)** | **P for Q-Test** | **P for Intercept** |
| --- | --- | --- | --- | --- |
| **Fasting glucose** |  |  |  |  |
| IVW | 0.479 | 0.998(0.991-1.004) | 1.35*10^-62^ | 0.251 |
| MR Egger | 0.185 | 0.998(0.971-1.006) |  |  |
| Simple mode | 0.885 | 0.999(0.988-1.010) |  |  |
| Weighted median | 0.799 | 1.001(0.996-1.005) |  |  |
| Weighted mode | 0.629 | 0.998(0.992-1.005) |  |  |
| **HbA1c** |  |  |  |  |
| IVW | 0.470 | 0.993(0.974-1.012) | 0.003 | 0.387 |
| MR Egger | 0.584 | 1.015(0.963-1.070) |  |  |
| Simple mode | 0.652 | 1.014(0.956-1.074) |  |  |
| Weighted median | 0.869 | 0.998(0.971-1.025) |  |  |
| Weighted mode | 0.969 | 1.001(0.961-1.042) |  |  |
| **T2DM** |  |  |  |  |
| IVW | 0.601 | 1.007(0.980-1.036) | 9.90*10^-47^ | 0.238 |
| MR Egger | 0.367 | 0.966(0.897-1.041) |  |  |
| Simple mode | 0.008 | 1.077(1.021-1.137) |  |  |
| Weighted median | 0.437 | 1.010(0.986-1.034) |  |  |
| Weighted mode | 0.247 | 1.019(0.987-1.053) |  |  |

T2DM, type 2 diseases; OR, odds ratio; IVW, inverse-variance weighted; Q-test: Cochran’s Q statistic;
