# Supplementary material for: No causal relationship between glucose and inflammatory bowel disease: a bidirectional two-sample mendelian randomization study
Source: BMC Med Genomics. 2024 Jun 12;17:159. doi: 10.1186/s12920-024-01923-6 (PMC11167808; doi:10.1186/s12920-024-01923-6)
Supplement: Supplementary file 2 — Supplementary Material 2 [file 12920_2024_1923_MOESM2_ESM.doc]

***Supplementary Material 2:*** ***Heterogeneity Plots***

**No Causal Relationship Between Glucose and** **Inflammatory Bowel Disease: A Bidirectional Two-Sample Mendelian Randomization Study**

JiePeng Cen, MD^1^†, Kequan Chen, MD^1^†, Ziyan Ni, MD^1^†, QiJie Dai^1^, MD, Weipeng Lu^1^, MD, Heqing Tao^1^, MD and Liang Peng^1^, MD

**Corresponding author:** Liang Peng: [wsfirefly@126.com](mailto:wsfirefly@126.com)

**1 Supplementary Figures**


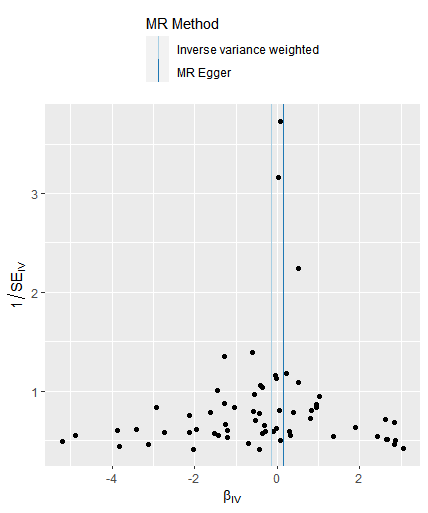


Figure 1.1 Heterogeneity plot where glucose as the exposure and inflammatory bowel disease (IBD) as the outcome.


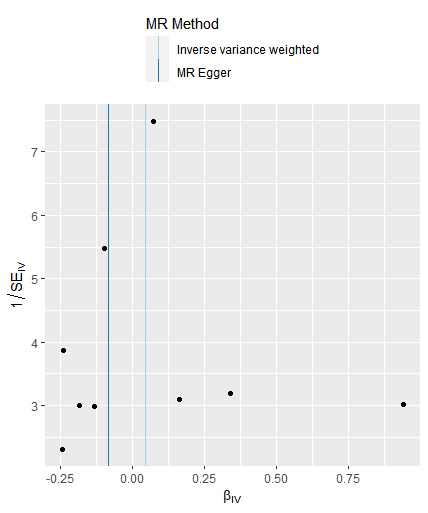


Figure 1.2 Heterogeneity plot where HbA1c as the exposure and IBD as the outcome.


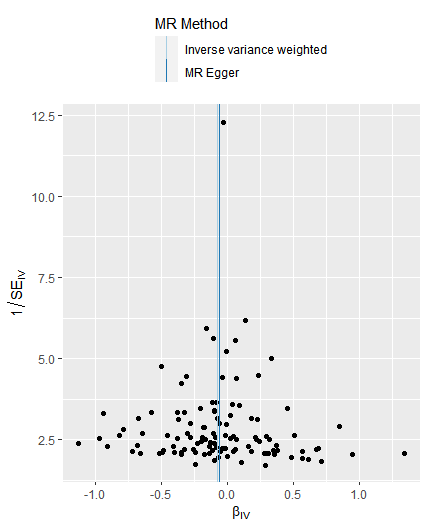


Figure 1.3 Heterogeneity plot where type 2 diabetes (T2DM) as the exposure and IBD as the outcome.


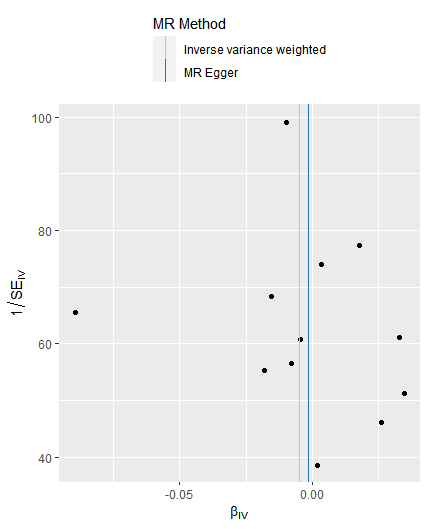


Figure 1.4 Heterogeneity plot where glucose as the outcome and IBD as the exposure.


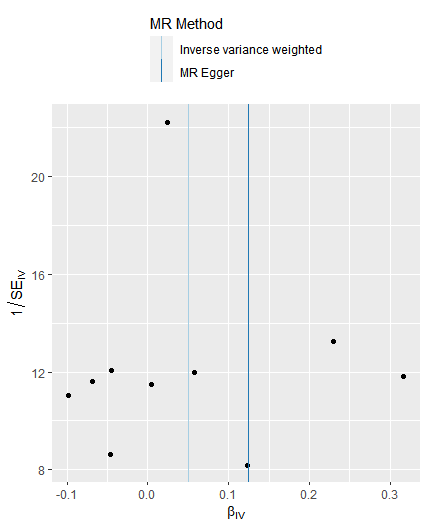


Figure 1.5 Heterogeneity plot where HbA1c as the outcome and IBD as the exposure.


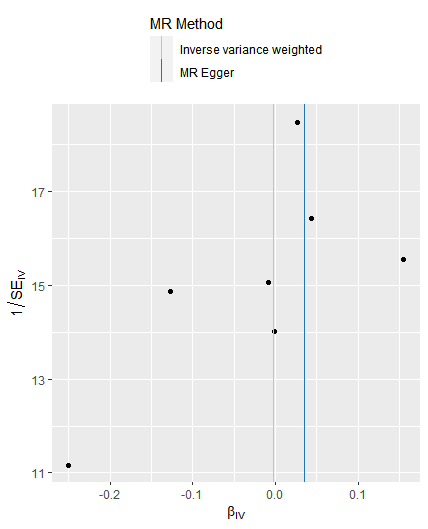


Figure 1.6 Heterogeneity plot where T2DM as the outcome and IBD as the exposure.


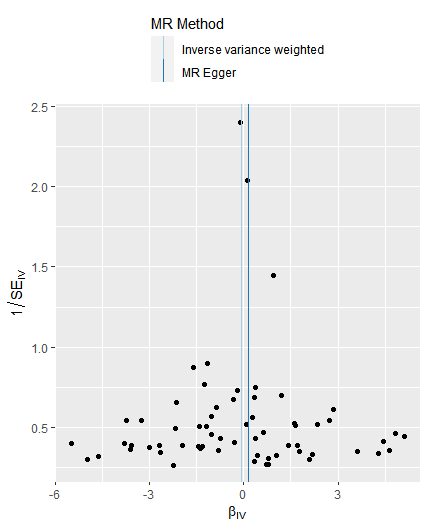


Figure 1.7 Heterogeneity plot where glucose as the exposure and ulcerative colitis (UC) as the outcome.


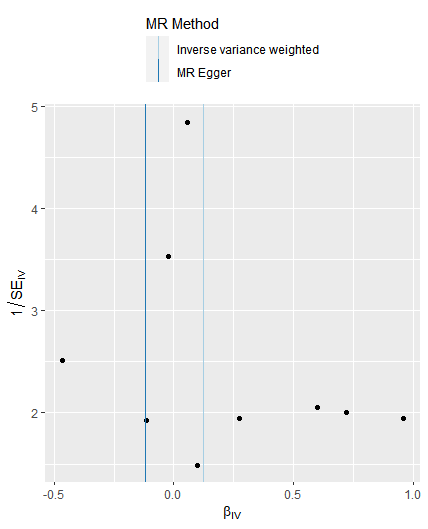


Figure 1.8 Heterogeneity plot where HbA1c as the exposure and UC as the outcome.


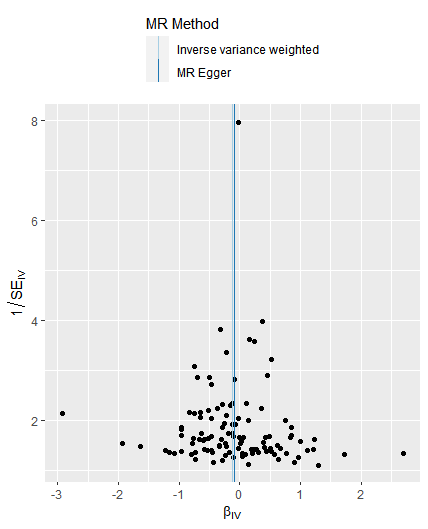


Figure 1.9 Heterogeneity plot where T2DM as the exposure and UC as the outcome.


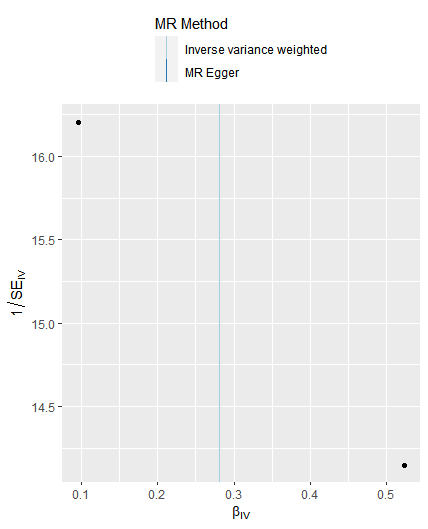


Figure 1.10 Heterogeneity plot where glucose as the outcome and UC as the exposure.


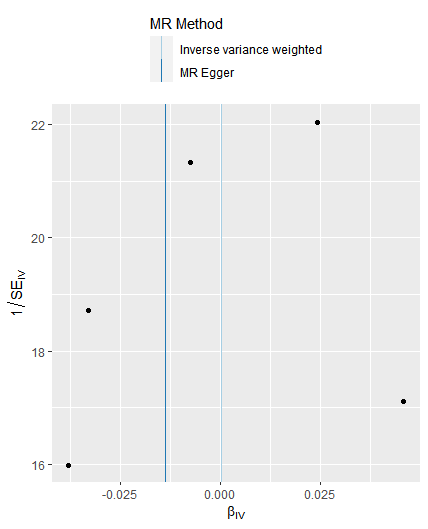


Figure 1.11 Heterogeneity plot where HbA1c as the outcome and UC as the exposure.


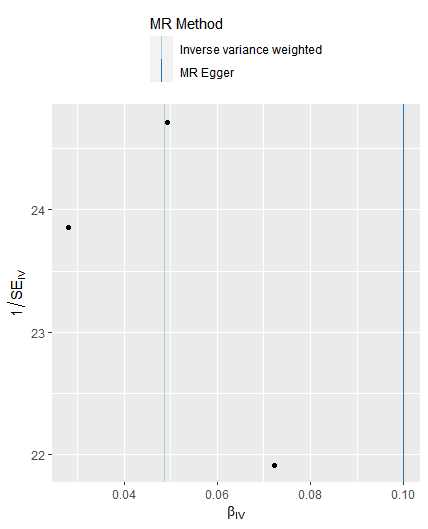


Figure 1.12 Heterogeneity plot where T2DM as the outcome and UC as the exposure.


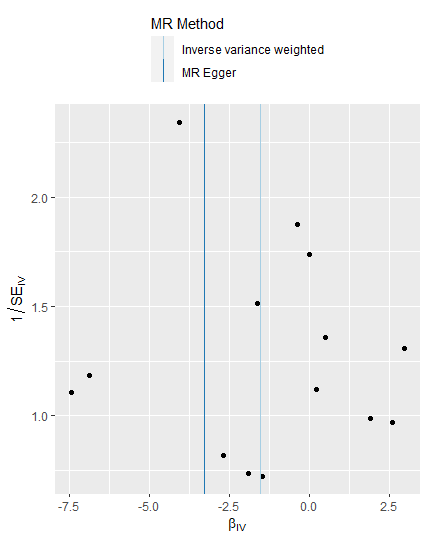


Figure 1.13 Heterogeneity plot where glucose as the exposure and Crohn’s disease (CD) as the outcome.


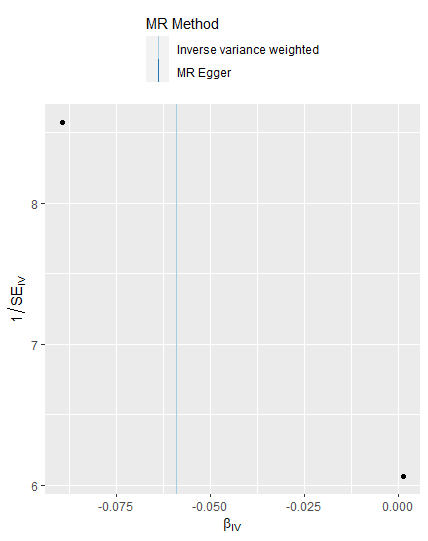


Figure 1.14 Heterogeneity plot where HbA1c as the exposure and CD as the outcome.


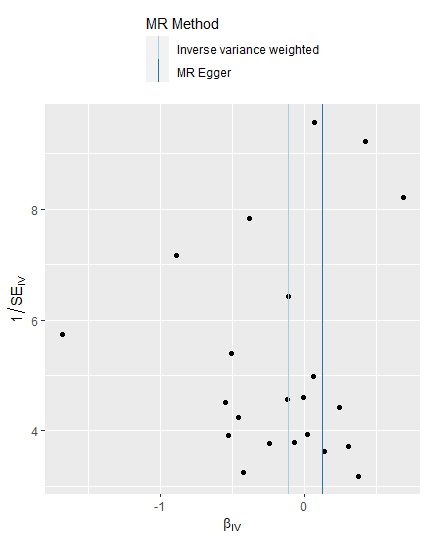


Figure 1.15 Heterogeneity plot where T2DM as the exposure and CD as the outcome.


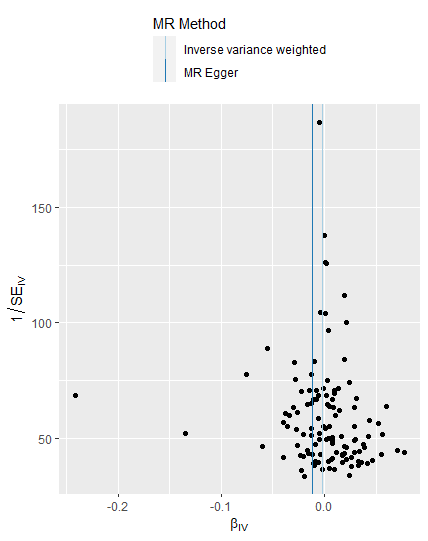


Figure 1.16 Heterogeneity plot where glucose as the outcome and CD as the exposure.


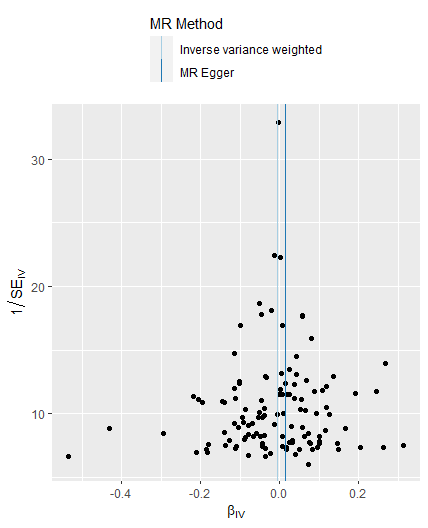


Figure 1.17 Heterogeneity plot where HbA1c as the outcome and CD as the exposure.


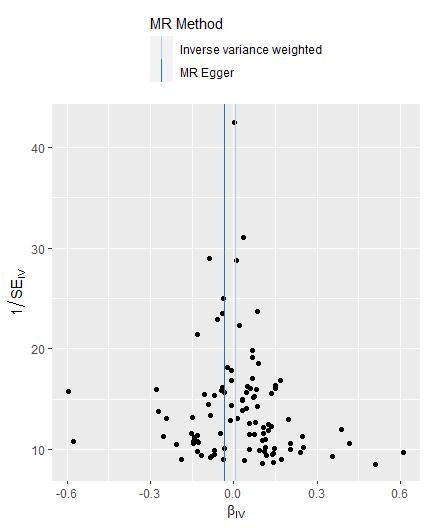


Figure 1.18 Heterogeneity plot where T2DM as the outcome and CD as the exposure.
