# Supplementary material for: No causal relationship between glucose and inflammatory bowel disease: a bidirectional two-sample mendelian randomization study
Source: BMC Med Genomics. 2024 Jun 12;17:159. doi: 10.1186/s12920-024-01923-6 (PMC11167808; doi:10.1186/s12920-024-01923-6)
Supplement: Supplementary file 3 — Supplementary Material 3 [file 12920_2024_1923_MOESM3_ESM.doc]

***Supplementary Material 3: Leave-one-out Plots***

**No Causal Relationship Between Glucose and** **Inflammatory Bowel Disease: A Bidirectional Two-Sample Mendelian Randomization Study**

JiePeng Cen, MD^1^†, Kequan Chen, MD^1^†, Ziyan Ni, MD^1^†, QiJie Dai^1^, MD, Weipeng Lu^1^, MD, Heqing Tao^1^, MD and Liang Peng^1^, MD

**Corresponding author:** Liang Peng: [wsfirefly@126.com](mailto:wsfirefly@126.com)

**1 Supplementary Figures**


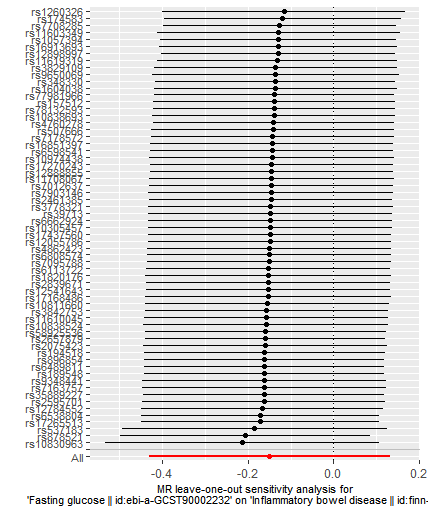


Figure 1.1 MR leave-one-out plot where glucose as the exposure and inflammatory bowel disease (IBD) as the outcome.


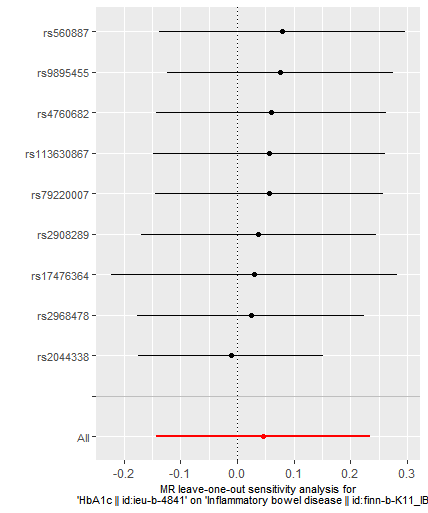


Figure 1.2 MR leave-one-out plot where HbA1c as the exposure and IBD as the outcome.


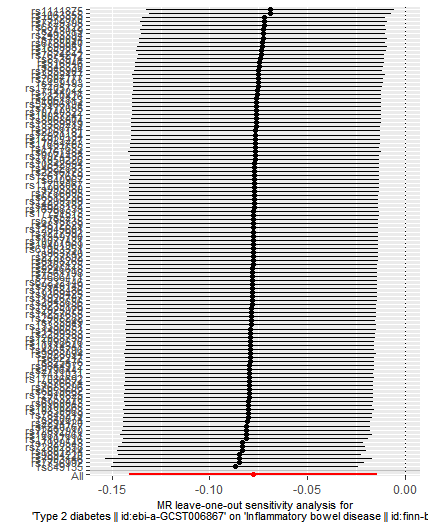


Figure 1.3 MR leave-one-out plot where type 2 diabetes (T2DM) as the exposure and IBD as the outcome.


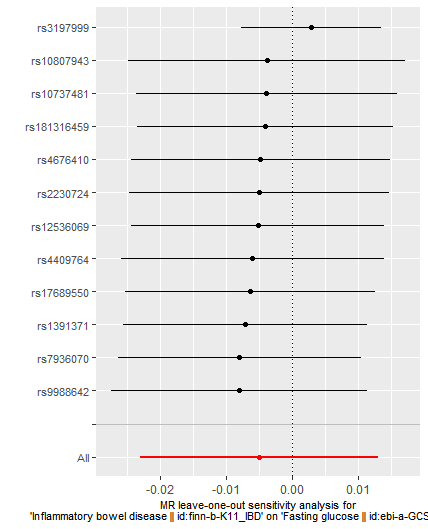


Figure 1.4 MR leave-one-out plot where glucose as the outcome and IBD as the exposure.


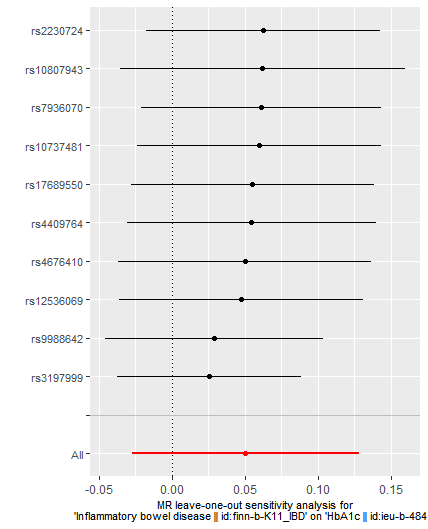


Figure 1.5 MR leave-one-out plot where HbA1c as the outcome and IBD as the exposure.


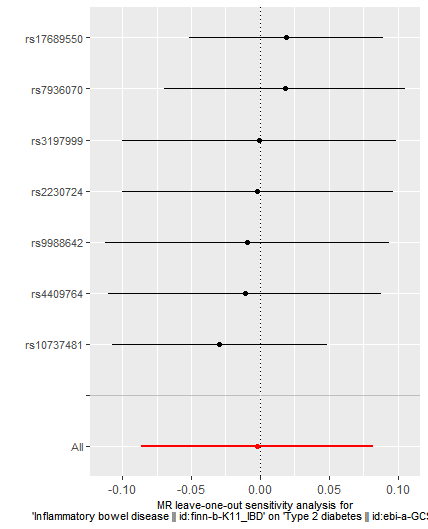


Figure 1.6 MR leave-one-out plot where T2DM as the outcome and IBD as the exposure.


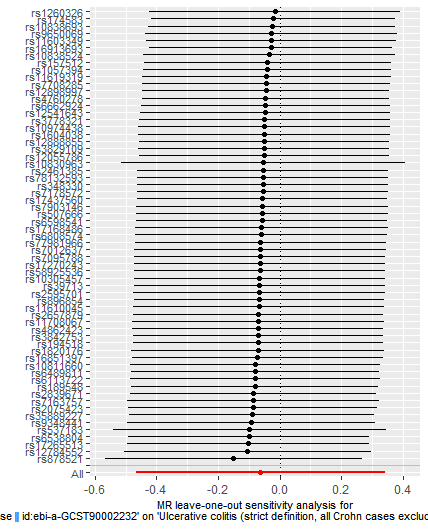


Figure 1.7 MR leave-one-out plot where glucose as the exposure and ulcerative colitis (UC) as the outcome.


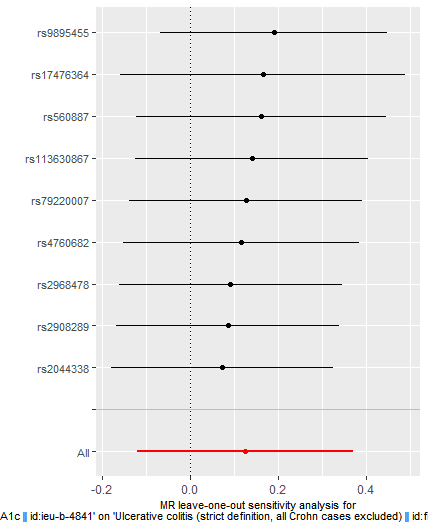


Figure 1.8 MR leave-one-out plot where HbA1c as the exposure and UC as the outcome.


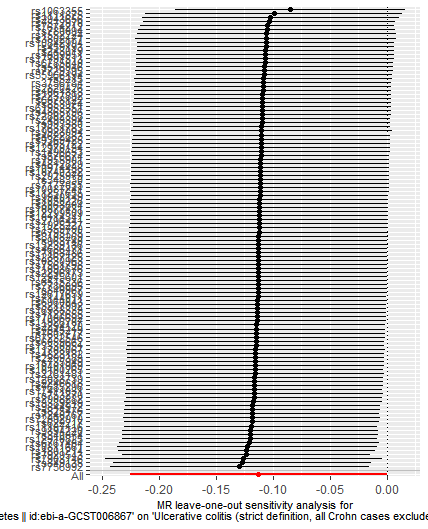


Figure 1.9 MR leave-one-out plot where T2DM as the exposure and UC as the outcome.


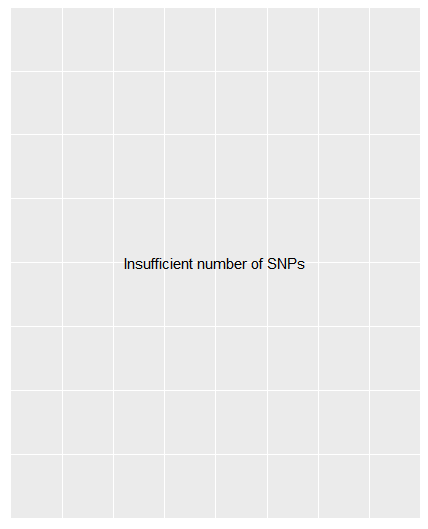


Figure 1.10 MR leave-one-out plot where glucose as the outcome and UC as the exposure.


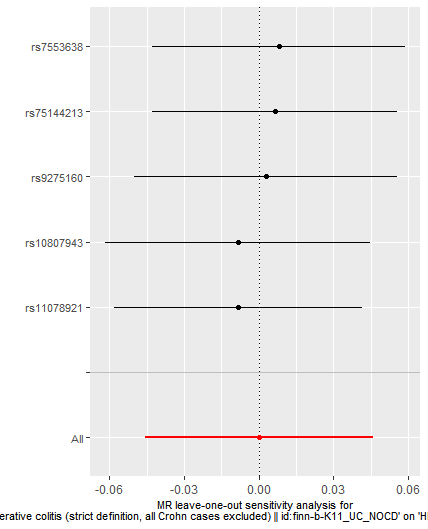


Figure 1.11 MR leave-one-out plot where HbA1c as the outcome and UC as the exposure.


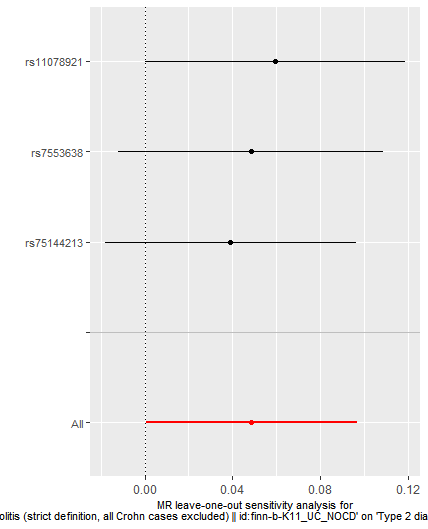


Figure 1.12 MR leave-one-out plot where T2DM as the outcome and UC as the exposure.


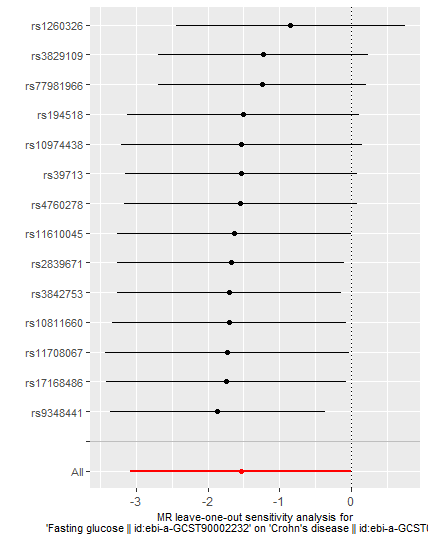


Figure 1.13 MR leave-one-out plot where glucose as the exposure and Crohn’s disease (CD) as the outcome.


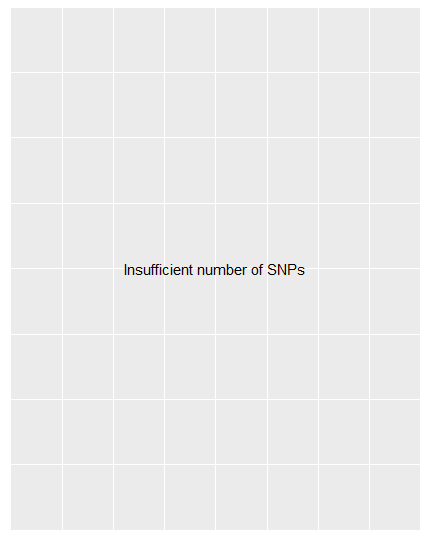


Figure 1.14 MR leave-one-out plot where HbA1c as the exposure and CD as the outcome.


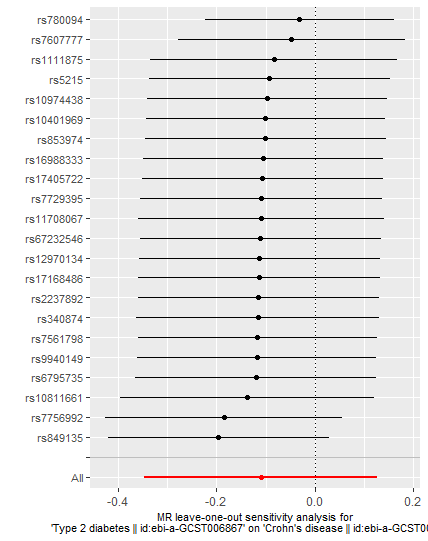


Figure 1.15 MR leave-one-out plot where T2DM as the exposure and CD as the outcome.


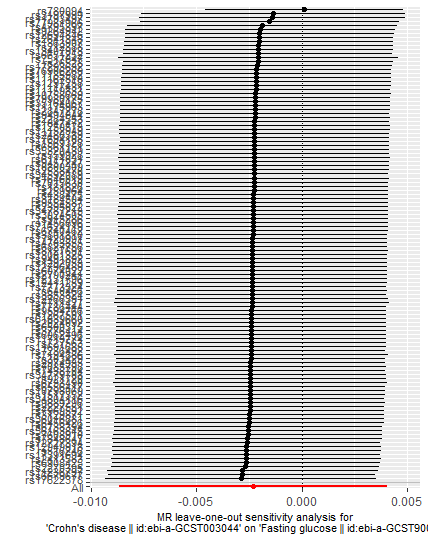


Figure 1.16 MR leave-one-out plot where glucose as the outcome and CD as the exposure.


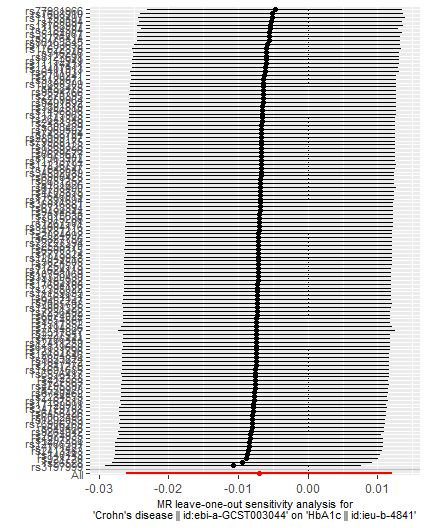


Figure 1.17 MR leave-one-out plot where HbA1c as the outcome and CD as the exposure.


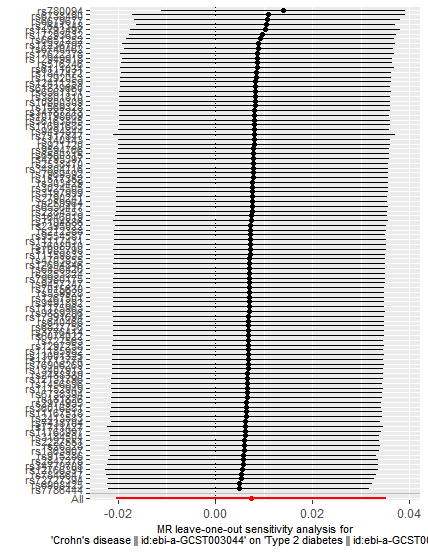


Figure 1.18 MR leave-one-out plot where T2DM as the outcome and CD as the exposure.
