# Supplementary material for: No causal relationship between glucose and inflammatory bowel disease: a bidirectional two-sample mendelian randomization study
Source: BMC Med Genomics. 2024 Jun 12;17:159. doi: 10.1186/s12920-024-01923-6 (PMC11167808; doi:10.1186/s12920-024-01923-6)
Supplement: Supplementary file 4 — Supplementary Material 4 [file 12920_2024_1923_MOESM4_ESM.doc]

***Supplementary Material 4: MR Results Scatter Plots***

**No Causal Relationship Between Glucose and** **Inflammatory Bowel Disease: A Bidirectional Two-Sample Mendelian Randomization Study**

JiePeng Cen, MD^1^†, Kequan Chen, MD^1^†, Ziyan Ni, MD^1^†, QiJie Dai^1^, MD, Weipeng Lu^1^, MD, Heqing Tao^1^, MD and Liang Peng^1^, MD

**Corresponding author:** Liang Peng: [wsfirefly@126.com](mailto:wsfirefly@126.com)

**1 Supplementary Figures**


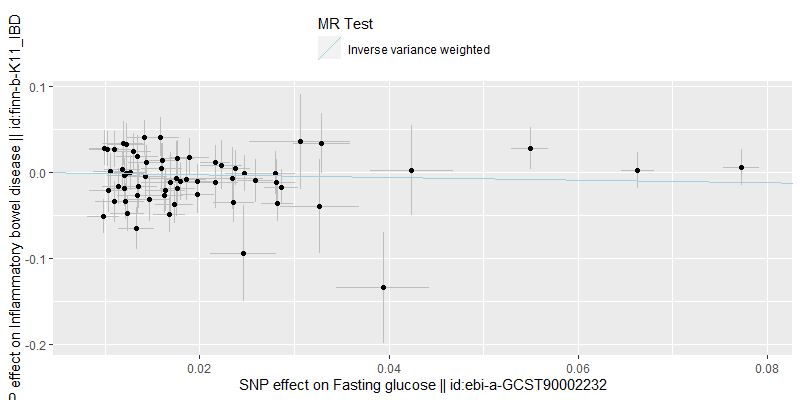


Figure 1.1 MR scatter plot where glucose as the exposure and IBD as the outcome. MR: Mendelian Randomization; IBD: Inflammatory Bowel Disease.


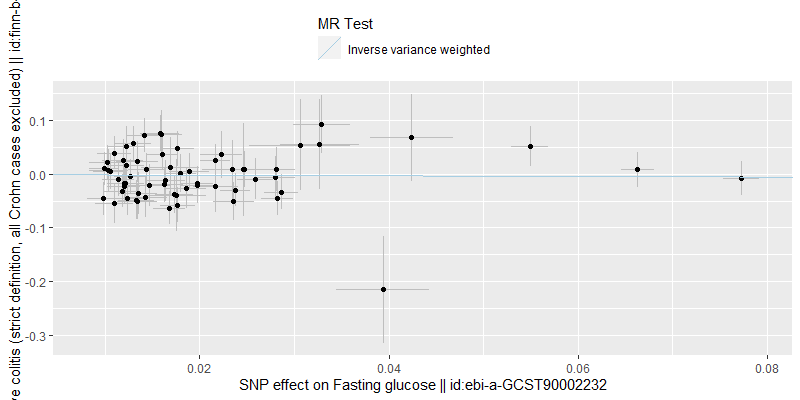


Figure 1.2 MR scatter plot where glucose as the exposure and UC as the outcome. MR: Mendelian Randomization; UC: ulcerative colitis


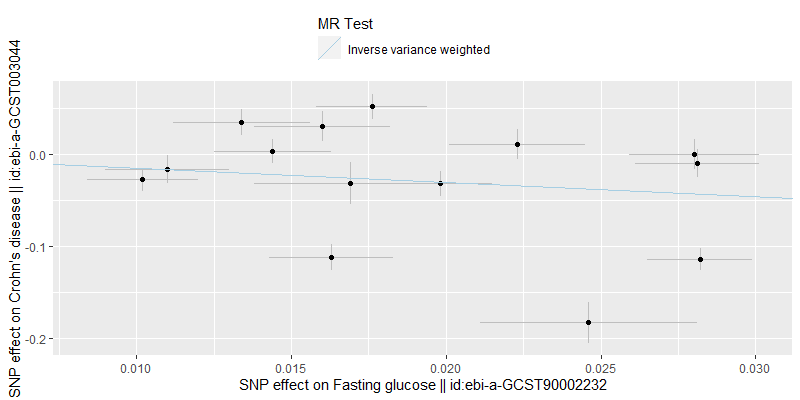


Figure 1.3 MR scatter plot where glucose as the exposure and CD as the outcome. MR: Mendelian Randomization; CD: Crohn’s disease.


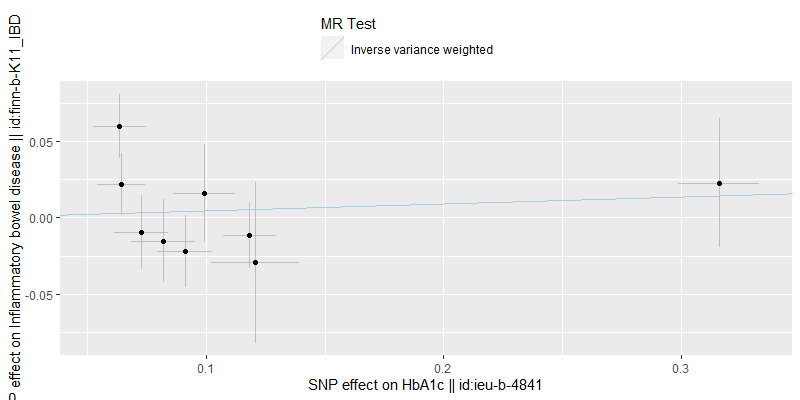


Figure 1.4 MR scatter plot where HbA1c as the exposure and IBD as the outcome. MR: Mendelian Randomization; IBD: Inflammatory Bowel Disease.


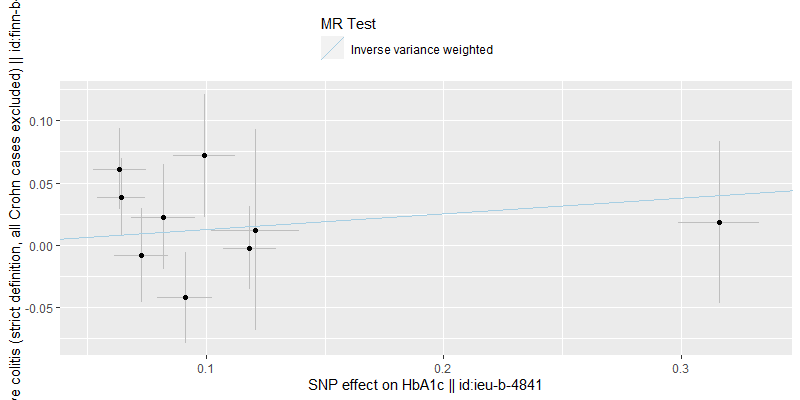


Figure 1.5 MR scatter plot where HbA1c as the exposure and UC as the outcome. MR: Mendelian Randomization; UC: ulcerative colitis.


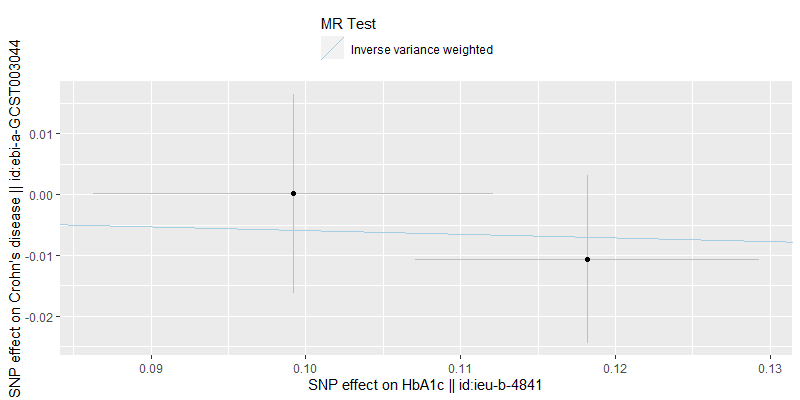


Figure 1.6 MR scatter plot where HbA1c as the exposure and CD as the outcome. MR: Mendelian Randomization; CD: Crohn’s disease.


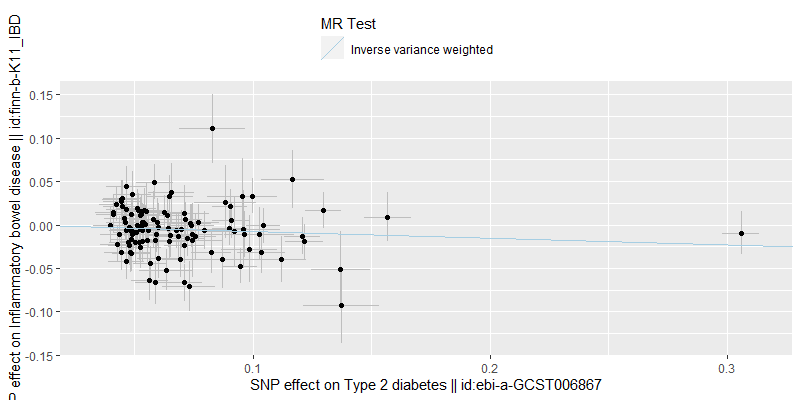


Figure 1.7 MR scatter plot where T2DM as the exposure and IBD as the outcome. MR: Mendelian Randomization; IBD: Inflammatory Bowel Disease; T2DM: type 2 diabetes.


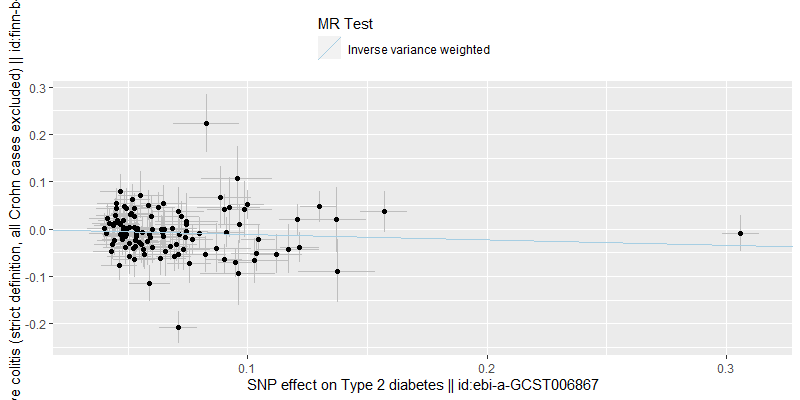


Figure 1.8 MR scatter plot where T2DM as the exposure and UC as the outcome. MR: Mendelian Randomization; UC: ulcerative colitis; T2DM: type 2 diabetes.


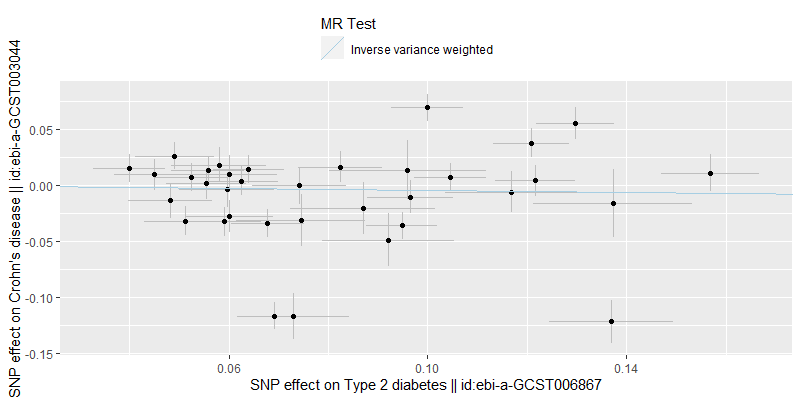


Figure 1.9 MR scatter plot where T2DM as the exposure and CD as the outcome. MR: Mendelian Randomization; CD: Crohn’s disease; T2DM: type 2 diabetes.


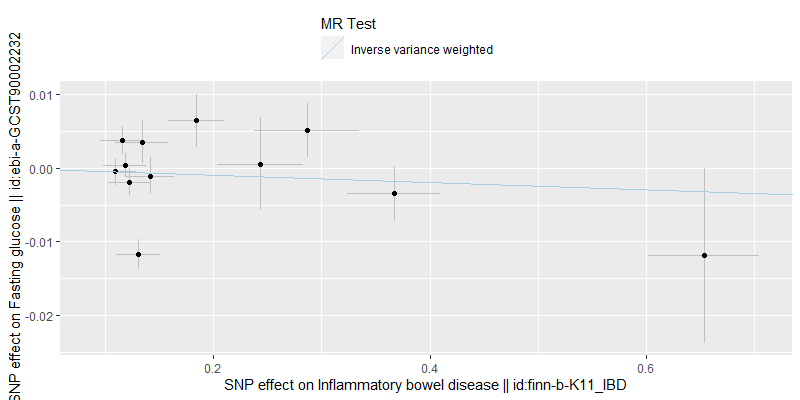


Figure 1.10 MR scatter plot where IBD as the exposure and glucose as the outcome. MR: Mendelian Randomization; IBD: Inflammatory Bowel Disease.


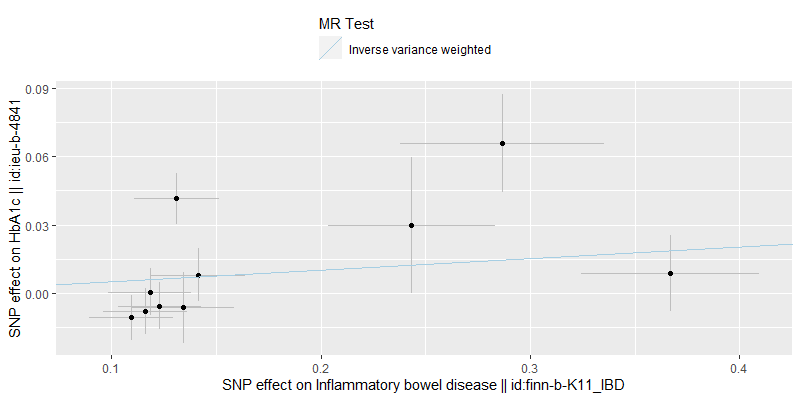


Figure 1.11 MR scatter plot where IBD as the exposure and HbA1c as the outcome. MR: Mendelian Randomization; IBD: Inflammatory Bowel Disease.


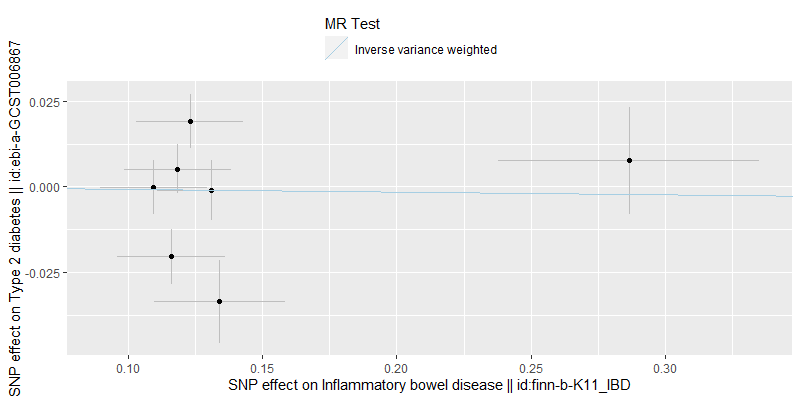


Figure 1.12 MR scatter plot where IBD as the exposure and T2DM as the outcome. MR: Mendelian Randomization; IBD: Inflammatory Bowel Disease; T2DM: type 2 diabetes.


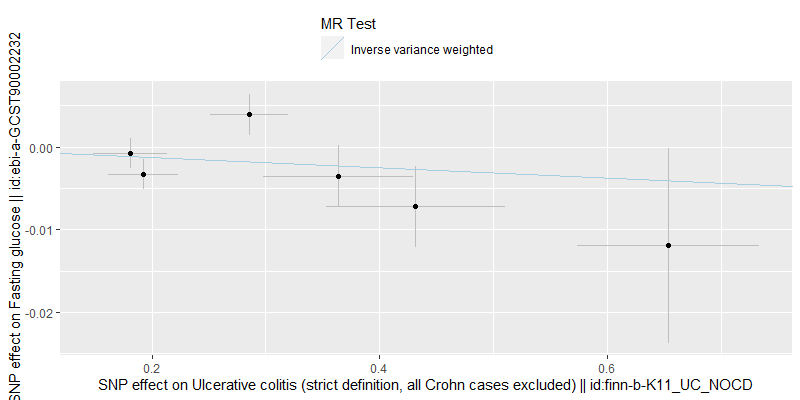


Figure 1.13 MR scatter plot where UC as the exposure and glucose as the outcome. MR: Mendelian Randomization; UC: ulcerative colitis.


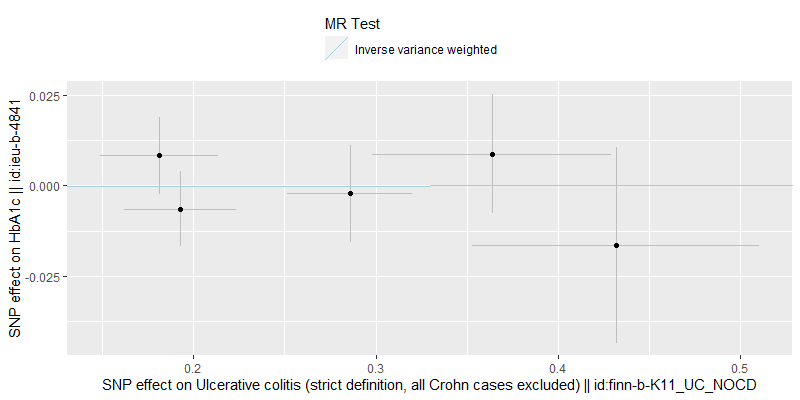


Figure 1.14 MR scatter plot where UC as the exposure and HbA1c as the outcome. MR: Mendelian Randomization; UC: ulcerative colitis.


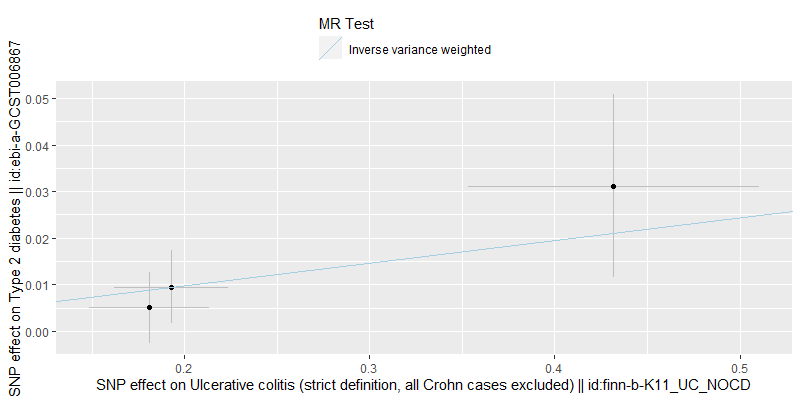


Figure 1.15 MR scatter plot where UC as the exposure and T2DM as the outcome. MR: Mendelian Randomization; UC: ulcerative colitis; T2DM: type 2 diabetes.


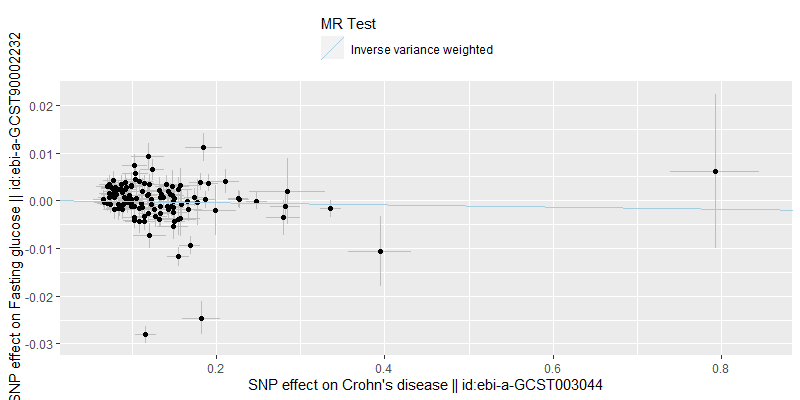


Figure 1.16 MR scatter plot where CD as the exposure and glucose as the outcome. MR: Mendelian Randomization; CD: Crohn’s disease.


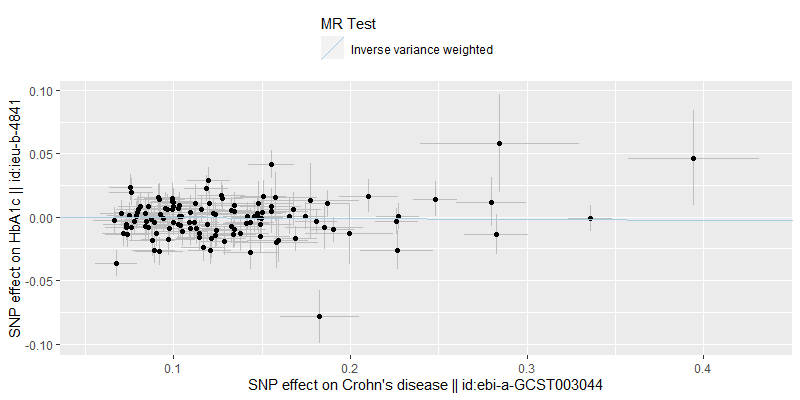


Figure 1.17 MR scatter plot where CD as the exposure and HbA1c as the outcome. MR: Mendelian Randomization; CD: Crohn’s disease.


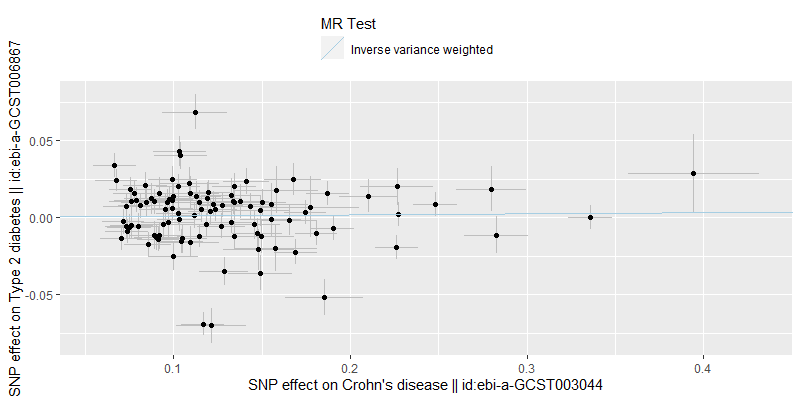


Figure 1.18 MR scatter plot where CD as the exposure and T2DM as the outcome. MR: Mendelian Randomization; CD: Crohn’s disease; T2DM: type 2 diabetes.
